# Supplementary material for: The interplay between social dominance and decision-making under expected and unexpected uncertainty: Evidence from event-related potentials
Source: PLoS One. 2025 Oct 17;20(10):e0334065. doi: 10.1371/journal.pone.0334065 (PMC12533924; doi:10.1371/journal.pone.0334065)
Supplement: S1 File — (ZIP) [file pone.0334065.s001.zip › S1_File.pdf]

PRF_d distribution

We first characterized the full PRF_d distribution across all 322 participants and then defined our final sample by selecting those at the extremes: individuals scoring between 1 and 7 comprised the low-dominance group, and those scoring between 11 and 16 comprised the high-dominance group (S1 Fig). To define "low" and "high" dominance groups, we first identified the extreme quartiles of the distribution, inviting those in the lowest quartile and the highest quartile to participate. We then continued inward from each tail—selecting additional individuals with the next-lowest and next-highest scores—until we reached our target of 30 low-dominance and 30 high-dominance participants.

S1 Table. PRF_d score distribution

Score	Frequency (n=322)	% (n=322)	Frequency (n=51)	% (n=51)
1	5	1.6%	0	0.0%
2	7	2.2%	1	2.0%
3	9	2.8%	1	2.0%
4	14	4.3%	4	7.8%
5	20	6.2%	5	9.8%
6	29	9.0%	7	13.7%
7	31	9.6%	5	9.8%
8	33	10.2%	0	0.0%
9	0	0.0%	0	0.0%
10	27	8.4%	0	0.0%
11	31	9.6%	8	15.7%
12	32	9.9%	6	11.8%
13	26	8.1%	7	13.7%
14	20	6.2%	3	5.9%

15	19	5.9%	3	5.9%
16	8	2.5%	1	2.0%

By juxtaposing the full 322-participant distribution against the 51-person subsample, we can see exactly what slice of the population was recruited and how “low” and “high” dominance map onto the empirical PRF-d curve. In the full cohort, scores 1–7 together account for 35.7% of participants, yet they represent 45.1% of our subsample (0% at 1, 2% at 2–3 each, then rising through 7). Conversely, scores 11–16 make up 42.2% of the full distribution but 55.0% of those selected (peaking at 15.7% for score 11 and tapering to 2% at 16). Meanwhile, the mids (8–10), which constitute 18.6% of the full sample, are entirely absent from the subsample. This enrichment of the bottom and top tails—and the complete exclusion of central scores—demonstrates that our “low” and “high” groups were explicitly defined as the extreme \~10–15% tails of the observed PRF-_d distribution, rather than arbitrary cutoff points.

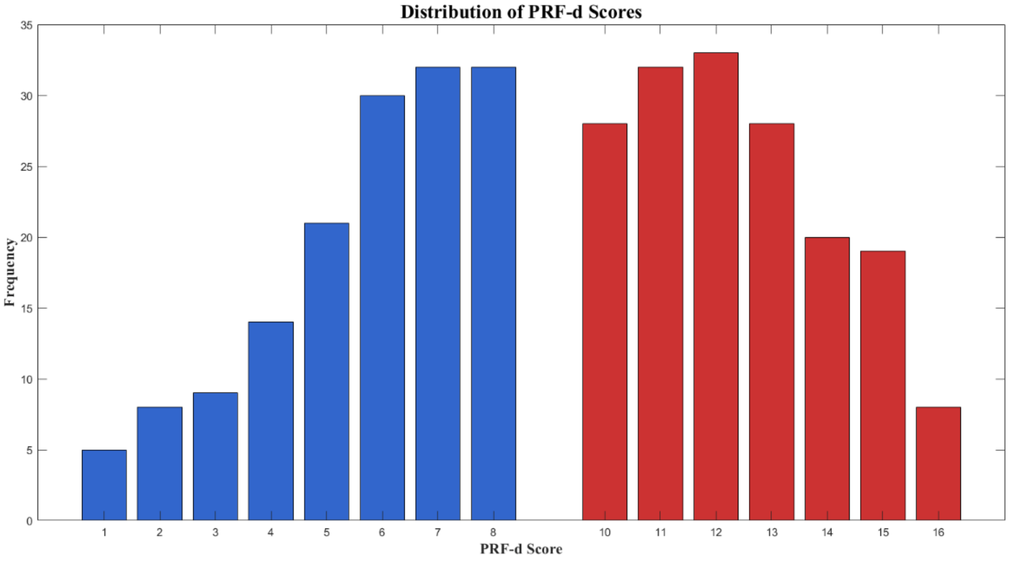

S1 Fig. PRF-d distribution
