# Supplementary material for: The interplay between social dominance and decision-making under expected and unexpected uncertainty: Evidence from event-related potentials
Source: PLoS One. 2025 Oct 17;20(10):e0334065. doi: 10.1371/journal.pone.0334065 (PMC12533924; doi:10.1371/journal.pone.0334065)
Supplement: S1 File — (ZIP) [file pone.0334065.s001.zip › S2_File.pdf]

## FRN amplitude

FRN amplitude was measured in three electrodes (Fz, FCz, and Cz). The following tests were performed independently on each electrode.

**Fz**

The ANOVA revealed a non-significant main effect for condition [$F_{(1.75, 85.63)} = 0.62$, $p = 0.920$, $\eta_p^2 = 0.001$], a significant main effect for valence [$F (1, 49) = 9.95$, $p = 0.003$, $\eta_p^2 = 0.169$], with higher FRN amplitude for negative than positive feedback, and a significant group effect [$F_{(1, 49)} = 14.69$, $p < 0.001$, $\eta_p^2 = 0.231$], with larger FRN amplitude in the low-dominance than high-dominance group. Moreover, significant interactions were observed between condition × valence [$F_{(2, 98)} = 5.68$, $p = 0.005$, $\eta_p^2 = 0.104$], and condition × group [$F_{(2, 98)} = 3.25$, $p = 0.043$, $\eta_p^2 = 0.062$]. No further interaction reached significance (all $F < 1.58$, $p > 0.210$).

The post-hoc analysis of the condition × valence interaction revealed no significant differences in the positive EXP-certain vs. EXP-uncertain (p = 0.865) and UNEXP-uncertain (p = 0.133) conditions, as well as in the EXP-uncertain vs. UNEXP-uncertain (p = 0.570) conditions. However, for negative valence, a significant difference was found between the EXP-certain vs. UNEXP-uncertain conditions (p = 0.001), with a larger FRN amplitude in the UNEXP-uncertain condition. There were no significant differences in the EXP-uncertain vs. EXP-certain (p = 0.742) and UNEXP-uncertain (p = 0.828) conditions. When examining individual conditions, both the EXP-certain (p = 0.929) and EXP-uncertain (p = 0.070) conditions showed comparable valence effects. However, the UNEXP-uncertain condition exhibited a larger FRN amplitude for negative feedback compared to positive feedback (p < 0.001).

To disentangle the condition × group interaction, when analysis separated by condition, a more negative FRN amplitude was found in low-dominance than high-dominance for all conditions (all Ps < 0.001). Other analyses did not reach statistical significance (all Ps > 0.308).

## FRN Latency

**Fz**

The ANOVA showed a significant main effect for valence [$F_{(1, 49)} = 4.03$, $p = 0.050$, $\eta_p^2 = 0.076$], with shorter FRN latency for negative than positive feedback, and a significant interaction between valence × group [$F_{(2, 98)} = 6.89$, $p = 0.012$, $\eta_p^2 = 0.123$]. No main effect or further interaction reached significance (all $F < 1.74$, $p > 0.181$).

The post-hoc analysis of the interaction between valence and the group showed that for the low-dominance group, the latency for negative feedback was shorter than that for positive feedback ($p = 0.003$), but this effect was not observed in the high-dominance group ($p = 0.648$). In terms of positive or negative valence, a marginally significant difference was observed ($p = 0.052$) between the low- and high-dominance groups for positive valence, where the low-dominance group had a longer FRN latency than the high-dominance group, but no significant effect was found for negative valence ($p = 0.726$) when comparing the two groups directly.
