# Supplementary material for: The interplay between social dominance and decision-making under expected and unexpected uncertainty: Evidence from event-related potentials
Source: PLoS One. 2025 Oct 17;20(10):e0334065. doi: 10.1371/journal.pone.0334065 (PMC12533924; doi:10.1371/journal.pone.0334065)
Supplement: S1 File — (ZIP) [file pone.0334065.s001.zip › S3_File.pdf]

FRN amplitude

FRN amplitude was measured in three electrodes (Fz, FCz, and Cz). The following tests were performed independently on each electrode.

FCz

The ANOVA showed a non-significant main effect for condition [$F_{(1.40, 68.58)} = 0.55$, $p = 0.516$, $\eta_p^2 = 0.011$], a significant main effect for valence [$F_{(1, 49)} = 8.91$, $p = 0.004$, $\eta_p^2 = 0.154$], with higher FRN amplitude for negative than positive feedback, and a significant main effect for group [$F_{(1, 49)} = 13.14$, $p < 0.001$, $\eta_p^2 = 0.212$], showing a larger FRN amplitude in the low-dominance than high-dominance group. There was also a significant interaction between condition × valence [$F_{(2, 98)} = 6.28$, $p = 0.003$, $\eta_p^2 = 0.114$]. No further interaction reached significance (all $F < 2.47$, $p > 0.122$) (S2 Fig).

Dividing the analysis by valence, the post-hoc results of the condition × valence interaction revealed for positive valence, there were no significant differences between the EXP-certain vs. EXP-uncertain, UNEXP-uncertain, and EXP-uncertain vs. UNEXP-uncertain conditions (all ps > 0.055). However, for negative valence, a significant difference was found between the EXP-certain vs. UNEXP-uncertain conditions ($p < 0.001$), where the UNEXP-uncertain condition had a larger FRN amplitude. There were no significant differences in the EXP-uncertain vs. EXP-certain ($p > 0.999$) and UNEXP-uncertain ($p = 0.372$) conditions. Upon examining individual conditions, both the EXP-certain ($p = 0.847$) and EXP-uncertain ($p = 0.179$) conditions exhibited comparable valence effects. However, the UNEXP-uncertain condition displayed a greater FRN amplitude for negative feedback than for positive feedback ($p < 0.001$).

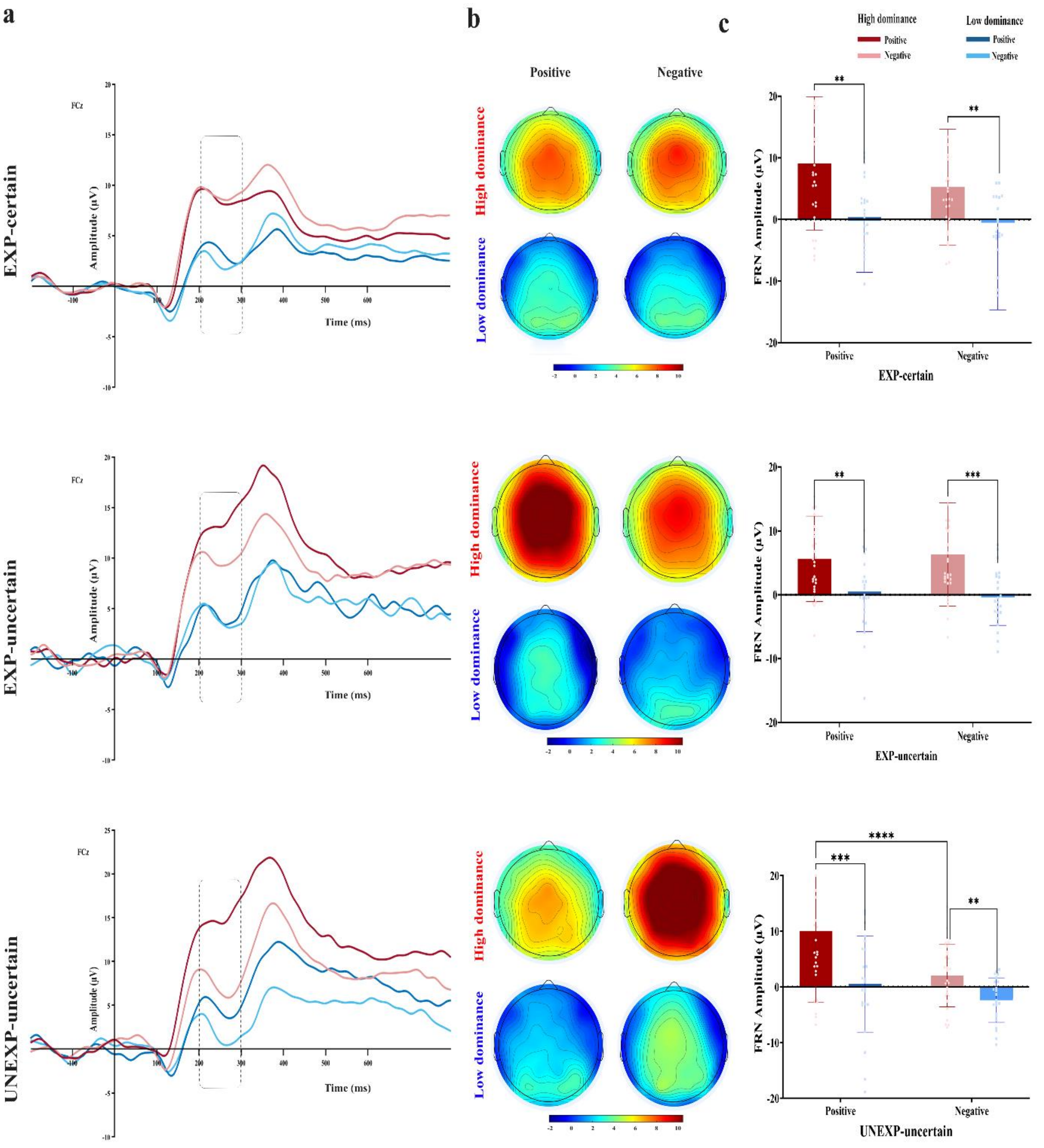

S2 Fig: FRN component in FCz electrode. a) Grand averaged ERP waveforms at Fz electrode for low and high dominance groups in positive, and negative feedback in three conditions (EXP-certain, EXP-uncertain, and UNEXP-uncertain). b) topographical scalp for the difference waves FRN component (200-300 ms post-feedback window) for each of the positive, and negative conditions for high and low dominance groups; in μV. C) FRN means amplitude differences; Error

bars denote SD. In all comparisons, "**" denotes $p < 0.01$, and "*" indicates $p < .05$ in all comparisons.

FRN Latency

FCZ

The ANOVA revealed a significant valence × group interaction [$F_{(1, 49)} = 8.22$, $p = 0.006$, $\eta_p^2 = 0.144$]. No main effect or further interaction reached significance (all $F < 3.24$, $p > 0.078$).

Upon dividing the participants into low- and high-dominance groups, the post-hoc comparison revealed that the low-dominance group demonstrated a shorter latency for negative feedback than for positive feedback ($p = 0.003$), while the high-dominance group did not exhibit a significant difference in latency based on valence ($p = 0.430$). When analyzing the data based on valence, a significant difference was observed between the two groups for positive feedback ($p = 0.021$), with the high-dominance group exhibiting a shorter latency than the low-dominance group. However, no significant difference was found between the low- and high-dominance groups for negative feedback ($p = 0.872$).
