# Supplementary material for: The interplay between social dominance and decision-making under expected and unexpected uncertainty: Evidence from event-related potentials
Source: PLoS One. 2025 Oct 17;20(10):e0334065. doi: 10.1371/journal.pone.0334065 (PMC12533924; doi:10.1371/journal.pone.0334065)
Supplement: S1 File — (ZIP) [file pone.0334065.s001.zip › S4_File.pdf]

FRN amplitude

FRN amplitude was measured in three electrodes (Fz, FCz, and Cz). The following tests were performed independently on each electrode.

Cz

The ANOVA showed a non-significant main effect for condition [$F_{(2, 98)} = 0.62$, $p = 0.940$, $\eta_p^2 = 0.011$], a significant main effect for valence [$F_{(1, 49)} = 11.64$, $p = 0.001$, $\eta_p^2 = 0.192$], with higher FRN amplitude for negative than positive feedback, and a significant main effect for group [$F_{(1, 49)} = 13.55$, $p = 0.001$, $\eta_p^2 = 0.217$], showing a larger FRN amplitude in the low-dominance than high-dominance group. There was also a significant interaction between condition × valence [$F_{(2, 98)} = 6.86$, $p = 0.002$, $\eta_p^2 = 0.123$]. No further interaction reached significance (all $F < 2.57$, $p > 0.116$) (S3 Fig).

When dividing the analysis by valence, the post-hoc results of the interaction between condition and valence showed that there were no significant differences between the EXP-certain vs. EXP-uncertain, UNEXP-uncertain, and EXP-uncertain vs. UNEXP-uncertain conditions (all ps > 0.090) for positive valence. However, for negative valence, a significant difference was observed between the EXP-certain vs. UNEXP-uncertain conditions ($p < 0.001$), where the UNEXP-uncertain condition exhibited a larger FRN amplitude. No significant differences existed between the EXP-uncertain vs. EXP-certain ($p = 0.506$) and UNEXP-uncertain ($p = 0.874$) conditions. Upon examining individual conditions, the EXP-certain condition showed comparable valence effects ($p = 0.847$). However, both the EXP-uncertain ($p = 0.013$) and UNEXP-uncertain ($p < 0.001$) conditions displayed a greater FRN amplitude for negative feedback compared to positive feedback.

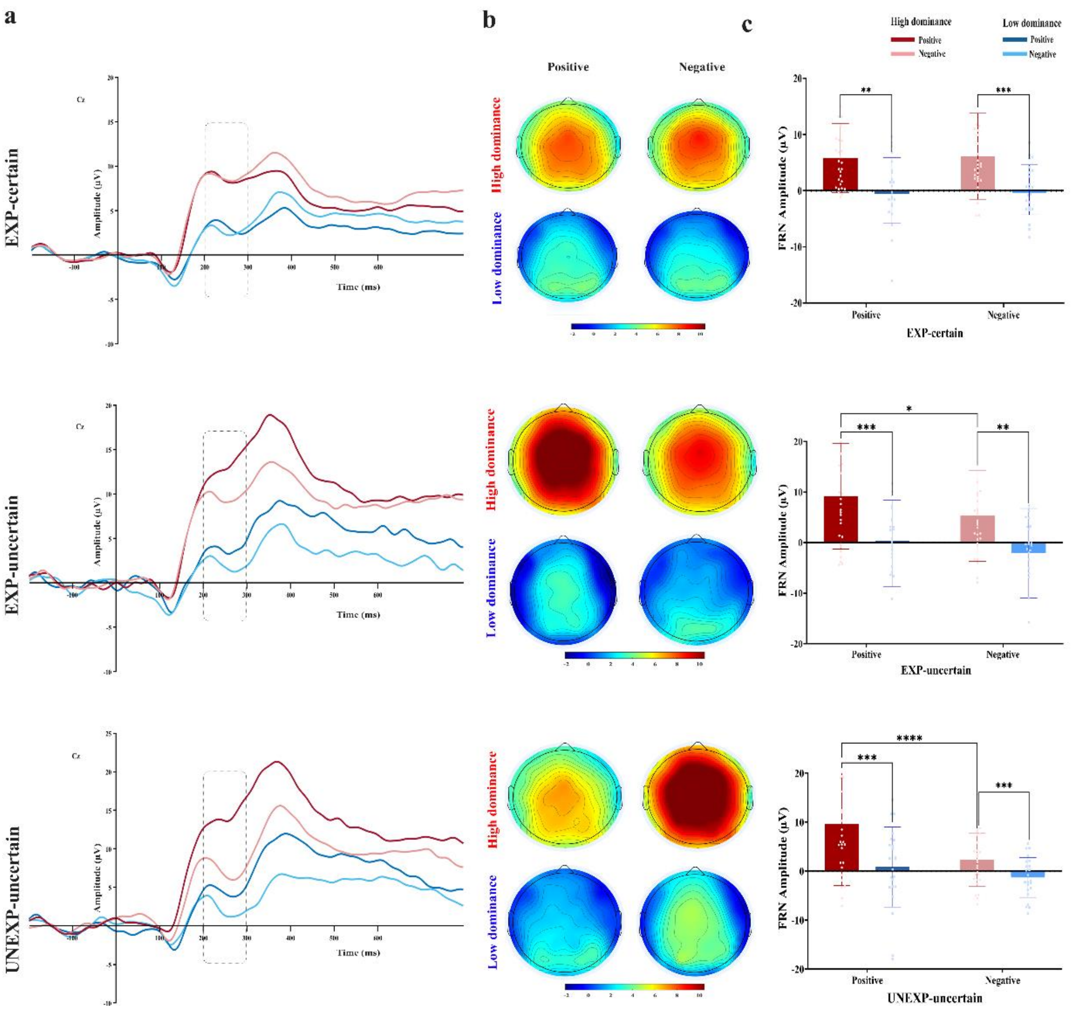

S3 Fig: FRN component in Cz electrode. a) Grand averaged ERP waveforms at Fz electrode for low and high dominance groups in positive, and negative feedback in three conditions (EXP-certain, EXP-uncertain, and UNEXP-uncertain). b) topographical scalp for the difference waves FRN component (200-300 ms post-feedback window) for each of the positive, and negative conditions for high and low dominance groups; in µV. C) FRN means amplitude differences; Error bars denote SD. In all comparisons, "**" denotes $p < 0.01$, and "*" indicates $p < .05$ in all comparisons.

FRN Latency

CZ

The ANOVA revealed a significant valence × group interaction [$F_{(1, 49)} = 5.37$, $p = 0.025$, $\eta_p^2 = 0.099$], and condition × valence interaction [$F_{(1.74, 85.63)} = 4.29$, $p = 0.021$, $\eta_p^2 = 0.081$]. No main effect or further interaction reached significance (all $F < 3.65$, $p > 0.062$).

After grouping participants separately, the post-hoc comparison revealed that the low-dominance group exhibited a shorter latency for negative feedback compared to positive feedback ($p = 0.006$), whereas the high-dominance group did not show a significant difference in latency between the two valences ($p = 0.763$). Moreover, when analyzing based on valence, a significant difference was observed for positive feedback between the two groups ($p = 0.021$), with the high-dominance group displaying shorter latency than the low-dominance group. However, for negative feedback, no significant difference was found between the low and high dominance groups ($p = 0.893$).

When dividing the analysis by valence, the post-hoc results of the interaction between condition and valence showed that there was a non-significant difference between the conditions within positive and negative valence (all $ps > 0.074$). Separating by condition, there was a significant difference between EXP-certain positive vs. EXP-certain negative conditions ($p = 0.001$), with shorter latency for negative than positive feedback. However, no significant differences were found when comparing positive and negative feedback in the EXP-uncertain and UNEXP-uncertain conditions (all $ps > 0.358$).
