# Supplementary material for: The interplay between social dominance and decision-making under expected and unexpected uncertainty: Evidence from event-related potentials
Source: PLoS One. 2025 Oct 17;20(10):e0334065. doi: 10.1371/journal.pone.0334065 (PMC12533924; doi:10.1371/journal.pone.0334065)
Supplement: Attachment — Submitted filename: response to reviewer plos1.docx [file pone.0334065.s002.docx]

**Author's Response to Editor and Reviewer Comments**

We are grateful to the reviewers for dedicating their time and attention to our manuscript. We believe that the manuscript has notably improved following the revisions made in response to their comments. In this document, original reviewer comments are presented in standard font, and dark blue color, while our responses are shown in italics, and black. Where applicable, modifications to the text are highlighted in bold.

I've reviewed the manuscript and agree with the concerns raised by the two reviewers regarding both the behavioral and neural data. The authors need to address these points thoroughly in their revision.

A critical improvement needed is the clear articulation of hypotheses in both behavioral and neural terms. Each hypothesis should be presented in a testable manner that establishes clear predictions. This clarity will significantly help readers understand the relationship between the behavioral findings and neural data patterns.

The manuscript would also benefit from stronger theoretical framing. Several ERP components discussed are traditionally explained within reinforcement learning frameworks, yet this theoretical perspective is underdeveloped in the current version. I recommend expanding the theoretical discussion to situate your findings within established models of reinforcement learning and to highlight the unique contributions your work makes to this literature.

These revisions will strengthen both the empirical foundation and theoretical significance of the work.

*First of all, thank you for the comments. Please find our answers to your insightful comments below:*

**Editor:**

1.Please ensure that your manuscript meets PLOS ONE's style requirements, including those for file naming. The PLOS ONE style templates can be found at

<https://journals.plos.org/plosone/s/file?id=wjVg/PLOSOne_formatting_sample_main_body.pdf> and

[https://journals.plos.org/plosone/s/file?id=ba62/PLOSOne_formatting_sample_title_authors_affiliations.pdf](https://track.editorialmanager.com/CL0/https:%2F%2Fjournals.plos.org%2Fplosone%2Fs%2Ffile%3Fid=ba62%2FPLOSOne_formatting_sample_title_authors_affiliations.pdf/1/010f01961015c1c5-4e38701d-ee9c-4ef6-af65-62c0c29acd2f-000000/pAVtkmoHQPnX1EpQ8plBFwMDiYk4aY3PUK02mBp-xTI=205)

***Answer****: Thank you for the reminder. I have reviewed the PLOS ONE style templates and updated my files to ensure that all formatting, headings, and file names comply with PLOS ONE style requirements.*

2**.** Thank you for stating the following financial disclosure:

“This project holds a scholarship entitled “TabrizU-300” by the International Academic Cooperation Directorate University of Tabriz, Iran.”

Please state what role the funders took in the study.  If the funders had no role, please state: "The funders had no role in study design, data collection and analysis, decision to publish, or preparation of the manuscript."

If this statement is not correct you must amend it as needed.

Please include this amended Role of Funder statement in your cover letter; we will change the online submission form on your behalf**.**

***Answer****: Thank you for your constructive feedback on the financial disclosure statement. We have revised the declaration on the role of the funders as follows*:

***Funding:*** *This project holds a scholarship entitled “TabrizU-300” by the International Academic Cooperation Directorate University of Tabriz, Iran.* ***The funders had no role in study design, data collection and analysis, decision to publish, or preparation of the manuscript.***

3. In this instance it seems there may be acceptable restrictions in place that prevent the public sharing of your minimal data. However, in line with our goal of ensuring long-term data availability to all interested researchers, PLOS’ Data Policy states that authors cannot be the sole named individuals responsible for ensuring data access (<http://journals.plos.org/plosone/s/data-availability#loc-acceptable-data-sharing-methods>).

Data requests to a non-author institutional point of contact, such as a data access or ethics committee, helps guarantee long term stability and availability of data. Providing interested researchers with a durable point of contact ensures data will be accessible even if an author changes email addresses, institutions, or becomes unavailable to answer requests.

Before we proceed with your manuscript, please also provide non-author contact information (phone/email/hyperlink) for a data access committee, ethics committee, or other institutional body to which data requests may be sent. If no institutional body is available to respond to requests for your minimal data, please consider if there any institutional representatives who did not collaborate in the study, and are not listed as authors on the manuscript, who would be able to hold the data and respond to external requests for data access? If so, please provide their contact information (i.e., email address). Please also provide details on how you will ensure persistent or long-term data storage and availability.

**Answer:** *Thank you for your valuable feedback and for highlighting the importance of long-term data access. In response, we have now uploaded all the required* *datasets underlying our findings to the Open Science Framework (OSF), a trusted and durable data repository that ensures persistent availability. The dataset can be accessed via the following link:* **“**<https://osf.io/4rsmq/>**”**

*We have updated the Data Availability statement in the revised manuscript accordingly and ensured that the data is publicly accessible through this platform. We believe this solution aligns with PLOS’s data sharing policy and provides long-term access for interested researchers.*
***page 44, Data availability: Data will be provided via the following link*** [***https://osf.io/4rsmg/***](https://osf.io/4rsmg/)***.***

4. Please include captions for your Supporting Information files at the end of your manuscript, and update any in-text citations to match accordingly. Please see our Supporting Information guidelines for more information: <http://journals.plos.org/plosone/s/supporting-information>.

***Answer:*** *Thank you for the suggestion. We have added captions for all Supporting Information files at the end of the manuscript and updated the corresponding in-text citations to match.*

***Page 56, Supporting information:***

***S1 Fig. PRF-d score distribution***

***S2 Fig. FRN component in Cz electrode. a) Grand averaged ERP waveforms at Fz electrode for low and high dominance groups in positive, and negative feedback in three conditions (EXP-certain, EXP-uncertain, and UNEXP-uncertain). b) topographical scalp for the difference waves FRN component (200-300 ms post-feedback window) for each of the positive, and negative conditions for high and low dominance groups; in μV. C) FRN means amplitude differences; Error bars denote SD. In all comparisons, "**" denotes p < 0.01, and "*" indicates p < .05 in all comparisons.***

***S3 Fig. FRN component in FCz electrode. a) Grand averaged ERP waveforms at Fz electrode for low and high dominance groups in positive, and negative feedback in three conditions (EXP-certain, EXP-uncertain, and UNEXP-uncertain). b) topographical scalp for the difference waves FRN component (200-300 ms post-feedback window) for each of the positive, and negative conditions for high and low dominance groups; in μV. C) FRN means amplitude differences; Error bars denote SD. In all comparisons, "**" denotes p < 0.01, and "*" indicates p < .05 in all comparisons.***

Additional Editor Comments:

I've reviewed the manuscript and agree with the concerns raised by the two reviewers regarding both the behavioral and neural data. The authors need to address these points thoroughly in their revision.

A critical improvement needed is the clear articulation of hypotheses in both behavioral and neural terms. Each hypothesis should be presented in a testable manner that establishes clear predictions. This clarity will significantly help readers understand the relationship between the behavioral findings and neural data patterns.

***Answer:*** *Thank you very much for your insightful feedback. We have edited the manuscript accordingly to clearly outline our hypotheses in behavioral and neural terms. Each hypothesis is now explicitly stated, with clear testable predictions about the modulation of behavioral sensitivity to feedback and neural response (i.e., FRN and P3 amplitudes) by social dominance under varying conditions of expectancy and uncertainty. This revision enhances the clarity of the relationship between our behavioral findings and neural data patterns, thereby facilitating better comprehension for readers.*

***Page 7-8, 1. Introduction, lines 153-174: The main question of this study was whether the amplitude and latency of the FRN and P3 components differ between high-dominance and low-dominance individuals when decision outcomes are either expected or unexpected. Using a well-established decision-making paradigm (12, 22), participants received positive and negative feedback under three feedback conditions—expected-certain, expected-uncertain, and unexpected-uncertain—while EEG recordings captured FRN and P3 activity. Based on prior research indicating that individuals with lower social dominance are more attuned to social evaluation and exhibit heightened sensitivity to external judgments in uncertain or hierarchical contexts (46, 49), we hypothesized that they would show more pronounced behavioral responses to negative feedback—particularly when outcomes are unexpected and uncertain—as a reflection of increased performance monitoring and concern for evaluation. At the neural level, we predicted that low-dominance individuals would exhibit larger FRN amplitudes in response to negative feedback under unexpected-uncertain conditions, indicating greater sensitivity to negative PEs. In contrast, we expected P3 amplitudes to be generally higher in response to unexpected-uncertain feedback than expected feedback across all participants, with high-dominance individuals showing larger P3 amplitudes overall due to their stronger motivational engagement and greater sensitivity to reward salience. Furthermore, to isolate neural correlates of RPE, we computed valence difference waves (negative minus positive feedback) for both FRN and P3 components (12, 22, 50-54). Given the evidence that high-dominance individuals exhibit stronger responses to reward cues and are more driven by reward-seeking behavior (49, 55-59), we expected more pronounced negative RPE signals in the unexpected-uncertain condition among high-dominance individuals relative to their low-dominance counterparts.***

The manuscript would also benefit from stronger theoretical framing. Several ERP components discussed are traditionally explained within reinforcement learning frameworks, yet this theoretical perspective is underdeveloped in the current version. I recommend expanding the theoretical discussion to situate your findings within established models of reinforcement learning and to highlight the unique contributions your work makes to this literature.

***Answer:*** *Thank you for your thoughtful and constructive feedback. We appreciate your suggestion to strengthen the theoretical framing. We expanded the introduction and discussion (At the comment of the second reviewer), and limitation in the manuscript to better highlight how our work contributes to this literature.*

***Page 3-8, 1. Introduction:*** *Humans face a tradeoff between exploration and exploitation when making decisions, which influences behavior from daily activities to occupational choices (1). Decision-making is a complex and challenging process, and the outcomes of decisions are often unclear and may lead to unfavorable results, requiring individuals to be mindful of uncertainty while making choices (2, 3). Uncertainty in decision-making comes in two forms: expected and unexpected. Expected uncertainty is the familiar unreliability of actions with probabilistic outcomes, like flipping a coin. Unexpected uncertainty, on the other hand, is a surprise – a sudden change in a previously reliable association, like a lever that always gave a reward but now doesn't. In other words, unexpected uncertainty arises from a violation of strong action-outcome links, while expected uncertainty stems from violations of weak, probabilistic ones (4).*

*In contexts characterized by uncertainty, efficient decision-making is crucial to minimize harm and optimize well-being and prompt assessment of the consequences of actions holds significant importance (5). Individuals heavily rely on feedback—whether positive or negative—to guide their future behavior. The brain likely employs specialized mechanisms to assess outcome value, magnitude, and to link feedback information with mental importance and motivation (6). This cognitive process is facilitated by distinct systems in the brain dedicated to processing rewards and losses (5).*

***Theoretical frameworks pertaining to reinforcement learning (RL) furnish a valuable paradigm for comprehending these neural mechanisms. RL theory asserts that learning occurs through iterative experimentation, wherein individuals assess the repercussions of their behaviors—actions yielding outcomes that exceed expectations are reinforced, whereas those resulting in outcomes that fall short of expectations are diminished. Central to this process is the concept of the prediction error (PE), which is the discrepancy between expected and actual outcomes, driving internal learning signals and expectation updates (7, 8).*** *One of the electroencephalographic components, known as the feedback-related negativity (FRN), is a well-established neural marker associated with loss processing (9). This negative deflection is typically observed 200-300 millisecond post-feedback onset in the frontal region, exhibits larger amplitudes in response to negative feedback compared to positive feedback (7, 9-16). Beyond simple outcome valence, the FRN is sensitive to factors such as uncertainty and unexpectedness (12****). Research suggests that the FRN reflects the encoding of PEs, aligning with RL theory (16).***

***Within the RL framework, the FRN is understood to reflect a rapid negative PE signal—often tied to mesencephalic dopamine signals—relayed to the anterior cingulate cortex (ACC), where it supports behavioral adaptation by identifying outcomes that are worse than predicted. The salience of this signal increases when outcomes are surprising or when uncertainty is high, making FRN a core index of adaptive learning (7, 17).*** *Studies by Yu et al. (2011) (18) and Pfabigan et al. (2011) (12) have shown that the FRN range is modulated by these factors, particularly for unexpected and uncertain outcomes. This suggests that the FRN may categorize outcomes as "good" or "bad" and signal "worse-than-expected" results via reward prediction error (RPE) signals. RPE represents the discrepancy between expected and actual outcomes, driving internal expectation updates (11, 19-23). The* *amplitude of RPE signals is influenced by outcome expectation, determined by factors such as certainty and likelihood. Mushtaq et al. (2011) (24)[22] defines certainty as the ability to accurately predict future events.*

*The P3 component plays a significant role in outcome evaluation and reward processing (25, 26). Following the detection of a mismatch between expected and actual results, the prediction model is updated to enhance its accuracy for future feedback. In response to reward and punishment stimuli, a positive deflection, known as P3, appears between 300 to 500 milliseconds post-feedback and is linked to this mechanism (27). Beyond its initial sensitivity to stimulus meaning and probability, the P3 component of the event-related potential (ERP) is essential in higher-order cognitive processes. It contributes to decision-making (27) and outcome evaluation (13), dynamically adapting its response to assess the functional significance of feedback stimuli (10, 28). This goes beyond mere performance tracking, reflecting the subjective significance and surprise associated with the stimuli (12, 29).*

***In RL terms, the P3 component is thought to reflect the allocation of attentional resources and motivational significance to feedback—particularly rewarding feedback. While the FRN signals an initial, automatic PE, the P3 is more involved in the consolidation and evaluation of the feedback's affective and motivational impact. Together, these components index distinct but complementary aspects of the RL feedback loop: immediate error detection (FRN) and the higher-order appraisal of outcome relevance (P3) (7, 17).***

*Furthermore, the P3 component demonstrates responsiveness to the absolute magnitude of rewards irrespective of the valence of the outcomes (positive or negative). This suggests a potential limitation in the evaluative function of feedback negativity, possibly restricting it to classifying events as either highly or lowly rewarding (30). Although examining brain activity provides insights into individual decision-making under uncertainty, a comprehensive understanding requires acknowledging various influencing factors. These include psychological aspects (e.g., biases and preferences) (31-33), and situational influences (e.g., social context and available information) (34-37). Social status, for example, can affect decision-making under both certain and uncertain conditions (37), with individuals sometimes altering their decisions when in conflicting social groups, whether consciously or unconsciously (38).*

***Importantly, recent theoretical advancements propose that RL processes transcend mere individual decision-making and may elucidate how individuals maneuver through intricate social landscapes, encompassing the formation and reinforcement of social dominance hierarchies. In this framework, dominance can be interpreted as an emergent characteristic of reward-oriented learning—individuals recalibrate their behavioral approaches based on perceived social rewards and penalties, acquiring knowledge regarding which behaviors engender deference or influence (7, 39).***

*Acquiring a dominant position within a hierarchy not only offers advantages but also facilitates the formation of effective social alliances by clearly indicating dominant relationships (40-42). Human social interactions are often marked by competition for hierarchical superiority. The pursuit of high status is conceptualized as dominance, a trait-like individual difference in personality and social psychology. Individuals vary in their propensity for dominance, with those showing higher levels more likely to achieve desired high-status positions (41). Behaviorally, dominance manifests as coercive control over resources, enabling individuals to command deference. Empirical evidence underscores the importance of dominance, distinguishing it from prestige, which relies on knowledge or shared benefits. Dominant individuals leverage their coercive power to gain social influence and preferential access to resources (43, 44).*

*Social dominance, signifying an individual's relative power and hierarchical position, exerts a demonstrable influence on decision-making processes. Individuals high in social dominance exhibit faster decision-making responses across various tasks, indicating a predisposition towards promptness in decision-making regardless of social context. Such activity has been associated with heightened brain function in regions including the insula, cingulate, right inferior temporal gyrus, and right angular gyrus (44). Moreover, individuals with elevated social status tend to prioritize rewarding outcomes, whereas those with lower status tend to assess outcomes in terms of potential risks and threats (45, 46).*

*While prior research on dominance mainly focused on behaviors within competitive social contexts, little is known about underlying individual traits (47, 48). This study posits that specific, context-independent traits of dominant individuals contribute to their social influence. Notably, unlike previous works that investigate how individual traits or manipulations modulate feedback processing (12, 18, 22), this study explores how social dominance levels lead to distinct neural and behavioral patterns for both positive and negative feedback across varying uncertainty levels. Therefore, our work aims to highlight how core feedback mechanisms—indexed by FRN and P3—are differentially modulated by stable personality traits like dominance, especially under varying levels of uncertainty. This provides new insights into how individuals learn to navigate complex, socially embedded environments using biologically grounded PE signals.*

*The main question of this study was whether the amplitude and latency of the FRN and P3 components differ between high-dominance and low-dominance individuals when decision outcomes are either expected or unexpected. Using a well-established decision-making paradigm (12, 22), participants received positive and negative feedback under three feedback conditions—expected-certain, expected-uncertain, and unexpected-uncertain—while EEG recordings captured FRN and P3 activity. Based on prior research indicating that individuals with lower social dominance are more attuned to social evaluation and exhibit heightened sensitivity to external judgments in uncertain or hierarchical contexts (46, 49), we hypothesized that they would show more pronounced behavioral responses to negative feedback—particularly when outcomes are unexpected and uncertain—as a reflection of increased performance monitoring and concern for evaluation. At the neural level, we predicted that low-dominance individuals would exhibit larger FRN amplitudes in response to negative feedback under unexpected-uncertain conditions, indicating greater sensitivity to negative PEs. In contrast, we expected P3 amplitudes to be generally higher in response to unexpected-uncertain feedback than expected feedback across all participants, with high-dominance individuals showing larger P3 amplitudes overall due to their stronger motivational engagement and greater sensitivity to reward salience. Furthermore, to isolate neural correlates of RPE, we computed valence difference waves (negative minus positive feedback) for both FRN and P3 components (12, 22, 50-54). Given the evidence that high-dominance individuals exhibit stronger responses to reward cues and are more driven by reward-seeking behavior (49, 55-59), we expected more pronounced negative RPE signals in the unexpected-uncertain condition among high-dominance individuals relative to their low-dominance counterparts.*

***Page 42-43, 4. Discussion, lines 931-945: Finally, our observations can profitably be interpreted in line with RL theory, which postulates that behavior is shaped by PEs (i.e., the difference between expected and actual outcome) that update the value attached to choice options through a learning rate parameter (95, 96). In this framework, FRN is widely considered to indicate a signed RPE, while the P3 component is thought to reflect the salience or associability of outcomes, scaling with the absolute magnitude of the PE (7, 52). In our study, both high- and low-dominance groups exhibited comparable behavioral learning trajectories during training—accuracy improvements and decreasing RTs—suggesting similar effective learning rates. A reflection of this is the pattern of the FRN amplitude, which was modulated by valence and expectancy of the outcome but did not differ between groups, implying matched computations of RPEs across levels of social dominance. However, the high-dominance group showed larger P3 amplitudes, especially with unexpected positive feedback, suggesting that there may be increased attentional engagement or salience attribution to rewarding outcomes. This dissociation matches RL models that argue for a separable role of RPE computation and outcome integration and suggests that dominance may modulate downstream processes related to outcome evaluation, rather than the very core error-driven learning mechanism.***

***Page 44, 4.3. Limitation, lines 964-967: Although we have not actually fitted RL models here, the behavioral and ERP results could be used to support or refute hypotheses in future modeling studies. For instance, extending this interpretation further, one might fit a Q-learning or RL-DDM model (reinforcement learning drift diffusion model) to choice and ERP data to estimate separate learning rates for gains and losses.***

**Reviewer #1**

This study aimed to unravel the neural and behavioral patterns associated with decision-making across varying social dominance levels under conditions of uncertainty. They investigated the properties of key neural correlates of feedback processing, including feedback-related negativity (FRN), and P3 components, and reward prediction error (RPE) signals. There are some suggestions.
*We appreciate the review and thank the reviewer for their time and attention to the manuscript. We have addressed the concerns below and modified the manuscript accordingly. We believe that these changes have helped improve the manuscript.*

The basis of the hypothesis is not clear, please revise the hypotheses parts, e.g. line 147“We expected that people with lower levels of dominance show more sensitivity to performance appraisal and more strongly evaluate their own performance”why did you get this hypothesis? and what is "more strongly evaluate their own performance"?

***Answer****: Thank you for your thoughtful and constructive feedback. We have revised the hypothesis section to clearly explain the theoretical basis of our predictions and clarified previously ambiguous phrasing to enhance precision and testability.*

***Page 7-8, 1. Introduction, lines 153-174: The main question of this study was whether the amplitude and latency of the FRN and P3 components differ between high-dominance and low-dominance individuals when decision outcomes are either expected or unexpected. Using a well-established decision-making paradigm (12, 22), participants received positive and negative feedback under three feedback conditions—expected-certain, expected-uncertain, and unexpected-uncertain—while EEG recordings captured FRN and P3 activity. Based on prior research indicating that individuals with lower social dominance are more attuned to social evaluation and exhibit heightened sensitivity to external judgments in uncertain or hierarchical contexts (46, 49), we hypothesized that they would show more pronounced behavioral responses to negative feedback—particularly when outcomes are unexpected and uncertain—as a reflection of increased performance monitoring and concern for evaluation. At the neural level, we predicted that low-dominance individuals would exhibit larger FRN amplitudes in response to negative feedback under unexpected-uncertain conditions, indicating greater sensitivity to negative PEs. In contrast, we expected P3 amplitudes to be generally higher in response to unexpected-uncertain feedback than expected feedback across all participants, with high-dominance individuals showing larger P3 amplitudes overall due to their stronger motivational engagement and greater sensitivity to reward salience. Furthermore, to isolate neural correlates of RPE, we computed valence difference waves (negative minus positive feedback) for both FRN and P3 components (12, 22, 50-54). Given the evidence that high-dominance individuals exhibit stronger responses to reward cues and are more driven by reward-seeking behavior (49, 55-59), we expected more pronounced negative RPE signals in the unexpected-uncertain condition among high-dominance individuals relative to their low-dominance counterparts.***

Line 96-126 is too long, please split it.

***Answer****: Thank you for your valuable feedback regarding the length and clarity of lines 96–126. To improve readability and organization, we have split the original passage into multiple paragraphs. Below, we provide the revised text with clearer segmentation, and we have included these changes in the updated manuscript.*

***Page 4-6, Introduction: The P3 component plays a significant role in outcome evaluation and reward processing [23, 24]. Following the detection of a mismatch between expected and actual results, the prediction model is updated to enhance its accuracy for future feedback. In response to reward and punishment stimuli, a positive deflection, known as P3, appears between 300 to 500 milliseconds post-feedback and is linked to this mechanism [25]. Beyond its initial sensitivity to stimulus meaning and probability, the P3 component of the event-related potential (ERP) is essential in higher-order cognitive processes. It contributes to decision-making [26] and outcome evaluation [11], dynamically adapting its response to assess the functional significance of feedback stimuli [8, 27]. This goes beyond mere performance tracking, reflecting the subjective significance and surprise associated with the stimuli [10, 28].***

***Furthermore, the P3 component demonstrates responsiveness to the absolute magnitude of rewards irrespective of the valence of the outcomes (positive or negative). This suggests a potential limitation in the evaluative function of feedback negativity, possibly restricting it to classifying events as either highly or lowly rewarding [29]. Although examining brain activity provides insights into individual decision-making under uncertainty, a comprehensive understanding requires acknowledging various influencing factors. These include psychological aspects (e.g., biases and preferences) [30–32] and situational influences (e.g., social context and available information) [33–36]. Social status, for example, can affect decision-making under both certain and uncertain conditions [36], with individuals sometimes altering their decisions when in conflicting social groups, whether consciously or unconsciously [37].***

***Acquiring a dominant position within a hierarchy not only offers advantages but also facilitates the formation of effective social alliances by clearly indicating dominant relationships [38–40]. Human social interactions are often marked by competition for hierarchical superiority. The pursuit of high status is conceptualized as dominance—a trait-like individual difference in personality and social psychology. Individuals vary in their propensity for dominance, with those showing higher levels more likely to achieve desired high-status positions [39]. Behaviorally, dominance manifests as coercive control over resources, enabling individuals to command deference. Empirical evidence underscores the importance of dominance, distinguishing it from prestige, which relies on knowledge or shared benefits. Dominant individuals leverage their coercive power to gain social influence and preferential access to resources [41, 42].***

In the training session it included only 60 trials, How can you be sure that these trials have led to subjects learning the characteristics of each cue?

***Answer****:* *Thank you for your valuable feedback. To ensure that the 60 training trials were sufficient for learning the characteristics of each cue, we conducted a pilot trial with three participants to confirm that the cue-response mappings could be learned within the allotted number of trials* ***(Page 13, 2.2.2. Decision-making task- lines 276-277****:* ***The paradigm was piloted with three participants to confirm that the cue-response mappings could be learned within the allotted number of trials).*** *Also, we implemented a verification process at the end of both the training and experimental sessions. Specifically, participants were asked to provide subjective estimates of the cue-feedback contingencies. These subjective estimates of positive feedback associated with each cue during the training session were compared with the actual positive feedback received in both sessions* ***(Figure 3)****. Additionally, any participant who did not demonstrate an adequate understanding of the instructions during the training session was excluded from further testing. This procedure has been described in the manuscript (****Page 11, 2.2.2. Decision-making task- lines 249-251: At the end of the training session, participants were asked about their subjective estimates of cue-feedback contingencies.*** ***Page 13,*** ***lines 272-275:*** ***At the end of the experiment, the participants were asked to estimate the probability of rewards for the three shapes for the training and experimental sessions to assess if the experimental manipulation was successful). Also, in the Results section (3.1.1 Positive feedback, and subjective estimates), we observed no significant differences between the Positive feedback, and subjective estimates. Therefore, during the training phase, participants effectively acquired the understanding of the cue-response relationship.***

***Also, While the training session included only 60 trials, multiple converging statistical results provide strong evidence that participants learned the characteristics of each cue during this phase. First, accuracy significantly increased from Block 1 to Block 2 for both groups and both cues, with Cue 1 improving from 74.55% to 91.96% and Cue 2 from 29.44% to 43.62% in the low-dominance group, and similar gains observed in the high-dominance group. These behavioral improvements were statistically confirmed by linear regressions, where all four cue–group combinations showed significant positive slopes (p-values ranging from 0.0006 to 0.0078), indicating systematic learning across trials. Additionally, mixed repeated-measures ANOVA revealed significant main effects for cue and block, as well as significant pre/post improvements in paired sample t-tests (all p < 0.001). Importantly, accuracy gains were accompanied by reductions in RTs, further supporting the presence of a learning effect. Despite the modest trial count, the consistency and strength of these results—across accuracy, RT, and multiple statistical tests—provide compelling evidence that learning occurred during the training session.***

***Page 20-24,*** ***3.1.3. Learning Rate***

***One behavioral consequence that can be interpreted through statistical analysis is the learning effect, particularly as reflected in changes in accuracy and RT during the training and Experiment phases across blocks. Fig 5 illustrates this trend by showing the average percentage of positive feedback, and RT shifts for each cue, separately for the low- and high-dominance groups.***

***The 60 training trials were divided into two blocks. In the low-dominance group, the mean percentage of positive feedback for Cue 1 increased from 74.55% in Block 1 to 91.96% in Block 2; for Cue 2, it rose from 29.44% to 43.62%. A similar trend was observed in the high-dominance group: Cue 1 increased from 76.85% to 92.65%, and Cue 2 from 25.72% to 40.19% (see Fig 5a).***

***Simple linear regression analyses confirmed statistically significant learning effects in all cues in both groups, with positive slopes indicating systematic improvements (high-dominance_cue1: 15.80 (p = 0.006), cue2: 14.47 (p < 0.001); low-dominance_cue1: 17.41 (p = 0.007), and cue2: 14.19 (p < 0.001)), each with modest but meaningful R² values (ranging from 0.13 to 0.29). Therefore, accuracy improved reliably with practice. Mixed repeated-measures ANOVA further supported these findings, revealing significant main effects for cue [F_(1, 49)_ = 252.39, p < 0.001,*** $\boldsymbol{\eta}_{\boldsymbol{p}}^{\boldsymbol{2}}$ ***= 0.837] and block [F_(1, 49)_ = 97.82, p < 0.001,*** $\boldsymbol{\eta}_{\boldsymbol{p}}^{\boldsymbol{2}}$***= 0.666], but not for group [F_(1, 49)_ = 0.14, p = 0.707,*** $\boldsymbol{\eta}_{\boldsymbol{p}}^{\boldsymbol{2}}$ ***= 0.003].***

***Paired samples t-tests revealed significant improvements across blocks for both groups. In the low-dominance group, Cue 1 rose from 74.55% to 91.96% and Cue 2 from 29.44% to 43.63% (both p < 0.001). Similarly, in the high-dominance group, Cue 1 improved from 76.85% to 92.65%, and Cue 2 from 25.72% to 40.19% (both p < 0.001). These findings suggest that both groups demonstrated comparable learning trajectories. Notably, the low-dominance group showed a slightly larger improvement for Cue 1 (17.41 vs. 15.80), although they started from a marginally lower baseline. For Cue 2, both groups improved by nearly the same magnitude, though the low-dominance group began with a higher initial accuracy. Moreover, the high-dominance group displayed less variability in Block 2, indicating more consistent performance.***

*In contrast, during the experiment session, no such learning trend emerged. For the low-dominance group, mean accuracy for Cue 1 increased slightly from 68.66% to 73.72%, while Cue 2 decreased marginally from 29.66% to 29.02%, and Cue 3 remained virtually unchanged (15.45% to 15.66%). The high-dominance group showed similar trends: Cue 1 increased from 70.07% to 74.68%, while Cue 2 and Cue 3 declined slightly (31.76% to 30.33% and 15.38% to 13.75%, respectively) (Fig. 5b). Linear regression analyses yielded non-significant slopes for all six conditions, indicating no meaningful learning effect across the blocks. Slopes ranged from –0.0884 to 0.3355, with all p > 0.1, and R² values near zero, suggesting stable performance without systematic improvement.*

*A mixed repeated-measures ANOVA during the experimental session confirmed a significant main effect for cue [F_(1.24, 60.86)_ = 1492.13, p < 0.001,* $\eta_{p}^{2}$*= 0.968], with higher accuracy for Cue 1 than Cues 2 and 3, and a significant main effect for block [F_(5.71, 279.86)_ = 3.3, p = 0.004,* $\eta_{p}^{2}$*= 0.063]. However, no significant main effect for group was found [F_(1, 49)_ = 1.20, p = 0.279,* $\eta_{p}^{2}$*= 0.024]. A significant interaction emerged between cue × block [F_(6.48, 317.59)_ = 2.77, p = 0.010,* $\eta_{p}^{2}$*= 0.054], while no other interactions reached significance.*

*Post-hoc analyses of the cue × block interaction revealed selective effects. For Cue 1, several blocks showed significant differences, suggesting minor fluctuations in performance. For example, Block 1 differed significantly from Block 5 (p = 0.049), and Block 3 from Block 8 (p = 0.002). Cue 2 exhibited only one significant difference (Block 2 vs. Block 5, p = 0.040), while Cue 3 showed two (Block 2 vs. Block 10, and Block 5 vs. Block 10, p < 0.001 and p = 0.006, respectively), indicating minimal variation over time.*

***In terms of RTs during the training session, both dominance groups showed general improvements. In the low-dominance group, RTs for Cue 1 declined from 0.73 to 0.65 seconds and for Cue 2 from 0.75 to 0.59. The high-dominance group displayed similar trends, with Cue 1 decreasing from 0.69 to 0.58 seconds and Cue 2 from 0.76 to 0.63 (Fig. 5c). Regression analysis revealed negative slopes for both cue 1 and cue 2 in both groups but only cue 2 in low-dominance group was statistically significant (R^2^ = 0.1403, f_(1,44)_ = 7.181, p = 0.010), suggesting that while all conditions trended downward, only one showed a robust practice-related RT improvement.***

***Mixed repeated-measures ANOVA on training RTs revealed a significant main effect of block [F_(1, 49)_ = 22.87, p < 0.001,*** $\boldsymbol{\eta}_{\boldsymbol{p}}^{\boldsymbol{2}}$***= 0.318], indicating faster responses in the second block. There were no significant main effects for cue [F _(1, 49)_ = 0.23, p = 0.630,*** $\boldsymbol{\eta}_{\boldsymbol{p}}^{\boldsymbol{2}}$ ***= 0.005]; and group [F _(1, 49)_ = 0.08, p = 0.775,*** $\boldsymbol{\eta}_{\boldsymbol{p}}^{\boldsymbol{2}}$ ***= 0.002]. Paired sample tests confirmed significant RT reductions in the high-dominance group for both cues (Cue 1: p = 0.002; Cue 2: p = 0.008). In contrast, only Cue 2 reached significance in the low-dominance group (p = 0.008), with Cue 1’s reduction falling short (p = 0.253). This suggests more consistent and generalized learning effects in the high-dominance group. RT variability was also higher in the low-dominance group, particularly for Cue 1, potentially accounting for the weaker effect.***

*During the experimental session, RTs also showed overall reductions, indicating a speed-accuracy trade-off. In the low-dominance group, Cue 1 decreased from 0.56 to 0.47 seconds and Cue 2 from 0.66 to 0.41, while Cue 3 slightly increased. In the high-dominance group, Cue 1 dropped from 0.58 to 0.46, Cue 2 from 0.70 to 0.52, and Cue 3 from 0.69 to 0.60 (Fig. 5d).*

*Simple linear regressions for RT during the experiment revealed significant negative slopes in cue 1, and cue 2 in both groups, (Slopes ranged from –0.01192 to –0.01853, all p value < 0.0025) again indicating modest but consistent learning effects. while cue 3 in low/high-dominance group remained non-significant (all P value > 0.21). Despite low R² values (low-dominance: –0.0056, high-dominance: –0.0020), these patterns confirm that repeated practice contributed to slight but reliable reductions in RT.*

*A mixed repeated-measures ANOVA of RT during the experiment confirmed a significant main effect for cue [F_(1.74, 85.63)_ = 32.24, p < 0.001,* $\eta_{p}^{2}$*= 0.397], with Cue 1 yielding the fastest responses. The main effect for block was also significant [F(6.31, 309.52) = 5.97, p < 0.001,* $\eta_{p}^{2}$*= 0.109], highlighting that responses were generally quicker by Block 10. No significant effect for group was found. Additionally, a significant interaction between cue and block emerged [F_(8.25, 404.71)_ = 2.61, p = 0.008,* $\eta_{p}^{2}$*= 0.051], with post-hoc comparisons confirming that Cue 1 showed the clearest pattern of decline over time (Block 1 vs. Block 4: p = 0.005; Block 1 vs. Block 8: p = 0.006; Block 1 vs. Block 9: p = 0.004; Block 2 vs. Block 4: p = 0.003; Block 2 vs. Block 6: p = 0.020; Block 2 vs. Block 7: p = 0.002; Block 2 vs. Block 8: p = 0.001; Block 2 vs. Block 9: p < 0.001; Block 2 vs. Block 10: p = 0.006) particularly between early and late blocks. Cue 2 displayed a similar, though less consistent (Block 1 vs. Block 7: p = 0.008; Block 1 vs. Block 8: p = 0.026; Block 1 vs. Block 9: p = 0.005; Block 1 vs. Block 10: p = 0.014), trend. Cue 3 remained largely stable across groups and blocks, with minimal changes.*

***Fig 5: The learning effect during the training and experiment phases across blocks, particularly as reflected in changes in accuracy (percentage of positive feedback) and RT; (a) left side: average of accuracy curves for cues in train session, right side: individual accuracy curves per cue and group in train session; (b) average of accuracy curves for cues in experiment session, right side: individual accuracy curves per cue and group in experiment session; (c) left side: RT in train session, right side: individual RT curves per cue and group in train session; (d) left side: RT in experiment session, right side: individual RT curves per cue and group in experiment session. Thick lines characterize the mean across task block and shaded areas indicate the standard error of the mean (sem).***

In the experimental session, the subjects will adjust their expectations according to the various results brought by the cue. The influence of the previous association in the training session may be effective only at the beginning, as the number of formal trials increases, the effect of this expectation will become less and less. How many of the 500 trials are affected by the expectation learnt in the training session? It is suggested that the researchers should analyze the whole experiment in sections and examine the characteristics of individual learning process.

***Answer****:* *We thank the reviewer for highlighting the value of a block‐wise analysis and have accordingly expanded the Result to clarify our approach. To pinpoint when training‐induced expectations wane, we examined both group‐level and individual learning curves. In brief, the training phase comprised two blocks of 30 trials each, and the main experiment comprised ten blocks of 50 trials each. Accuracy and reaction time (RT) were analyzed for each cue type (Cue 1, Cue 2, Cue 3) within low‐ and high‐dominance groups (****Page 20-24, 3.1.3. Learning Rate****).*

*To pinpoint when training‐induced expectations wane, we examined both group‐level and individual learning curves. Early blocks (approximately the first 200–250 trials, or Blocks 4-5) showed evidence of expectation‐driven performance: elevated accuracy and faster RTs for previously trained cues, coupled with significant cue × block interactions. Thereafter, these effects diminished as participants progressively relied on new feedback rather than prior associations. Notably, some low‐dominance participants maintained high accuracy for Cue 1 through Block 4, whereas high‐dominance participants converged on the experiment’s contingencies more rapidly, typically by Block 3.*

***Page 20-24, 3.1.3. Learning Rate: One behavioral consequence that can be interpreted through statistical analysis is the learning effect, particularly as reflected in changes in accuracy and RT during the training and Experiment phases across blocks. Fig 5 illustrates this trend by showing the average percentage of positive feedback, and RT shifts for each cue, separately for the low- and high-dominance groups.***

***The 60 training trials were divided into two blocks. In the low-dominance group, the mean percentage of positive feedback for Cue 1 increased from 74.55% in Block 1 to 91.96% in Block 2; for Cue 2, it rose from 29.44% to 43.62%. A similar trend was observed in the high-dominance group: Cue 1 increased from 76.85% to 92.65%, and Cue 2 from 25.72% to 40.19% (see Fig. 5a).***

***Simple linear regression analyses confirmed statistically significant learning effects in all cues in both groups, with positive slopes indicating systematic improvements (high-dominance_cue1: 15.80 (p = 0.006), cue2: 14.47 (p < 0.001); low-dominance_cue1: 17.41 (p = 0.007), and cue2: 14.19 (p < 0.001)), each with modest but meaningful R² values (ranging from 0.13 to 0.29). Therefore, accuracy improved reliably with practice. Mixed repeated-measures ANOVA further supported these findings, revealing significant main effects for cue [F_(1, 49)_ = 252.39, p < 0.001,*** $\boldsymbol{\eta}_{\boldsymbol{p}}^{\boldsymbol{2}}$ ***= 0.837] and block [F_(1, 49)_ = 97.82, p < 0.001,*** $\boldsymbol{\eta}_{\boldsymbol{p}}^{\boldsymbol{2}}$***= 0.666], but not for group [F_(1, 49)_ = 0.14, p = 0.707,*** $\boldsymbol{\eta}_{\boldsymbol{p}}^{\boldsymbol{2}}$ ***= 0.003].***

***Paired samples t-tests revealed significant improvements across blocks for both groups. In the low-dominance group, Cue 1 rose from 74.55% to 91.96% and Cue 2 from 29.44% to 43.63% (both p < 0.001). Similarly, in the high-dominance group, Cue 1 improved from 76.85% to 92.65%, and Cue 2 from 25.72% to 40.19% (both p < 0.001). These findings suggest that both groups demonstrated comparable learning trajectories. Notably, the low-dominance group showed a slightly larger improvement for Cue 1 (17.41 vs. 15.80), although they started from a marginally lower baseline. For Cue 2, both groups improved by nearly the same magnitude, though the low-dominance group began with a higher initial accuracy. Moreover, the high-dominance group displayed less variability in Block 2, indicating more consistent performance.***

***In contrast, during the experiment session, no such learning trend emerged. For the low-dominance group, mean accuracy for Cue 1 increased slightly from 68.66% to 73.72%, while Cue 2 decreased marginally from 29.66% to 29.02%, and Cue 3 remained virtually unchanged (15.45% to 15.66%). The high-dominance group showed similar trends: Cue 1 increased from 70.07% to 74.68%, while Cue 2 and Cue 3 declined slightly (31.76% to 30.33% and 15.38% to 13.75%, respectively) (Fig. 5b). Linear regression analyses yielded non-significant slopes for all six conditions, indicating no meaningful learning effect across the blocks. Slopes ranged from –0.0884 to 0.3355, with all p > 0.1, and R² values near zero, suggesting stable performance without systematic improvement.***

***A mixed repeated-measures ANOVA during the experimental session confirmed a significant main effect for cue [F_(1.24, 60.86)_ = 1492.13, p < 0.001,*** $\boldsymbol{\eta}_{\boldsymbol{p}}^{\boldsymbol{2}}$***= 0.968], with higher accuracy for Cue 1 than Cues 2 and 3, and a significant main effect for block [F_(5.71, 279.86)_ = 3.3, p = 0.004,*** $\boldsymbol{\eta}_{\boldsymbol{p}}^{\boldsymbol{2}}$***= 0.063]. However, no significant main effect for group was found [F_(1, 49)_ = 1.20, p = 0.279,*** $\boldsymbol{\eta}_{\boldsymbol{p}}^{\boldsymbol{2}}$***= 0.024]. A significant interaction emerged between cue × block [F_(6.48, 317.59)_ = 2.77, p = 0.010,*** $\boldsymbol{\eta}_{\boldsymbol{p}}^{\boldsymbol{2}}$***= 0.054], while no other interactions reached significance.***

***Post-hoc analyses of the cue × block interaction revealed selective effects. For Cue 1, several blocks showed significant differences, suggesting minor fluctuations in performance. For example, Block 1 differed significantly from Block 5 (p = 0.049), and Block 3 from Block 8 (p = 0.002). Cue 2 exhibited only one significant difference (Block 2 vs. Block 5, p = 0.040), while Cue 3 showed two (Block 2 vs. Block 10, and Block 5 vs. Block 10, p < 0.001 and p = 0.006, respectively), indicating minimal variation over time.***

***In terms of RTs during the training session, both dominance groups showed general improvements. In the low-dominance group, RTs for Cue 1 declined from 0.73 to 0.65 seconds and for Cue 2 from 0.75 to 0.59. The high-dominance group displayed similar trends, with Cue 1 decreasing from 0.69 to 0.58 seconds and Cue 2 from 0.76 to 0.63 (Fig. 5c). Regression analysis revealed negative slopes for both cue 1 and cue 2 in both groups but only cue 2 in low-dominance group was statistically significant (R^2^ = 0.1403, f_(1,44)_ = 7.181, p = 0.010), suggesting that while all conditions trended downward, only one showed a robust practice-related RT improvement.***

***Mixed repeated-measures ANOVA on training RTs revealed a significant main effect of block [F_(1, 49)_ = 22.87, p < 0.001,*** $\boldsymbol{\eta}_{\boldsymbol{p}}^{\boldsymbol{2}}$***= 0.318], indicating faster responses in the second block. There were no significant main effects for cue [F _(1, 49)_ = 0.23, p = 0.630,*** $\boldsymbol{\eta}_{\boldsymbol{p}}^{\boldsymbol{2}}$ ***= 0.005]; and group [F _(1, 49)_ = 0.08, p = 0.775,*** $\boldsymbol{\eta}_{\boldsymbol{p}}^{\boldsymbol{2}}$ ***= 0.002]. Paired sample tests confirmed significant RT reductions in the high-dominance group for both cues (Cue 1: p = 0.002; Cue 2: p = 0.008). In contrast, only Cue 2 reached significance in the low-dominance group (p = 0.008), with Cue 1’s reduction falling short (p = 0.253). This suggests more consistent and generalized learning effects in the high-dominance group. RT variability was also higher in the low-dominance group, particularly for Cue 1, potentially accounting for the weaker effect.***

***During the experimental session, RTs also showed overall reductions, indicating a speed-accuracy trade-off. In the low-dominance group, Cue 1 decreased from 0.56 to 0.47 seconds and Cue 2 from 0.66 to 0.41, while Cue 3 slightly increased. In the high-dominance group, Cue 1 dropped from 0.58 to 0.46, Cue 2 from 0.70 to 0.52, and Cue 3 from 0.69 to 0.60 (Fig. 5d).***

***Simple linear regressions for RT during the experiment revealed significant negative slopes in cue 1, and cue 2 in both groups, (Slopes ranged from –0.01192 to –0.01853, all p value < 0.0025) again indicating modest but consistent learning effects. while cue 3 in low/high-dominance group remained non-significant (all P value > 0.21). Despite low R² values (low-dominance: –0.0056, high-dominance: –0.0020), these patterns confirm that repeated practice contributed to slight but reliable reductions in RT.***

***A mixed repeated-measures ANOVA of RT during the experiment confirmed a significant main effect for cue [F_(1.74, 85.63)_ = 32.24, p < 0.001,*** $\boldsymbol{\eta}_{\boldsymbol{p}}^{\boldsymbol{2}}$***= 0.397], with Cue 1 yielding the fastest responses. The main effect for block was also significant [F(6.31, 309.52) = 5.97, p < 0.001,*** $\boldsymbol{\eta}_{\boldsymbol{p}}^{\boldsymbol{2}}$***= 0.109], highlighting that responses were generally quicker by Block 10. No significant effect for group was found. Additionally, a significant interaction between cue and block emerged [F_(8.25, 404.71)_ = 2.61, p = 0.008,*** $\boldsymbol{\eta}_{\boldsymbol{p}}^{\boldsymbol{2}}$***= 0.051], with post-hoc comparisons confirming that Cue 1 showed the clearest pattern of decline over time (Block 1 vs. Block 4: p = 0.005; Block 1 vs. Block 8: p = 0.006; Block 1 vs. Block 9: p = 0.004; Block 2 vs. Block 4: p = 0.003; Block 2 vs. Block 6: p = 0.020; Block 2 vs. Block 7: p = 0.002; Block 2 vs. Block 8: p = 0.001; Block 2 vs. Block 9: p < 0.001; Block 2 vs. Block 10: p = 0.006) particularly between early and late blocks. Cue 2 displayed a similar, though less consistent (Block 1 vs. Block 7: p = 0.008; Block 1 vs. Block 8: p = 0.026; Block 1 vs. Block 9: p = 0.005; Block 1 vs. Block 10: p = 0.014), trend. Cue 3 remained largely stable across groups and blocks, with minimal changes.***

***Fig 5: The learning effect during the training and experiment phases across blocks, particularly as reflected in changes in accuracy (percentage of positive feedback) and RT; (a) left side: average of accuracy curves for cues in train session, right side: individual accuracy curves per cue and group in train session; (b) average of accuracy curves for cues in experiment session, right side: individual accuracy curves per cue and group in experiment session; (c) left side: RT in train session, right side: individual RT curves per cue and group in train session; (d) left side: RT in experiment session, right side: individual RT curves per cue and group in experiment session. Thick lines characterize the mean across task block and shaded areas indicate the standard error of the mean (sem).***

Why only three conditions in the experiment? 1) expected-certain feedback, 2) expected-uncertain feedback, and 3) unexpected uncertain feedback, Why not design unexpected certain conditions？
***Answer****:* *Thank you for this insightful comment. We agree that the conceptual space of expectancy and certainty provides a larger array of possible combinations. Nonetheless, our paradigm was purposely designed to look at the neural correlations of violated expectation under conditions of uncertainty, not just at any sort of expectancy-certainty combination. In our task, we focused on the following three feedback conditions:*

*1. Expected-certain feedback (e.g., 80% positive feedback for cue1, 80% negative feedback for cue3),*

*2. Expected-uncertain feedback (e.g., 50% positive/negative feedback for cue2), and*

*3. Unexpected-uncertain feedback (e.g., 20% unexpected negative feedback for cue1, 20% unexpected positive feedback for cue3).*

*These conditions were selected because they represent the most informative contrasts for our theoretical aims, which were to explore:*

*- how participants maintain learned expectations despite probabilistic violations, and*

*- how the brain responds when such expectations are violated under uncertainty.*

*We did not include an "unexpected-certain" feedback condition; because, by definition, certainty implies predictability—making it logically incompatible with unexpected outcomes. For example, if feedback is delivered with 100% consistency, then by nature it cannot violate expectations (i.e., it is not "unexpected"). Conversely, if an outcome is unexpected, it must occur in a context where some level of uncertainty exists (i.e., the participant has an expectation that is probabilistically violated). Thus, while theoretically interesting, an "unexpected-certain" condition would be conceptually contradictory in the context of our probabilistic learning task.*

It could be a very important result to test the correlation among personality scores, brain electricity, and behavior to investigate the explanation path of personality-brain mechanism-behavior.
***Answer****:* *Thank you for this insightful suggestion. We agree that examining the relationships between personality traits, brain activity, and behavioral outcomes can offer important insights into the personality–brain–behavior pathway. In response, we have conducted additional correlation analyses among PRF-d scores, EEG amplitudes, and behavioral RTs. We added the results of these analyses to the revised manuscript in the results and the discussion sections, which will further enlighten us on the mechanistic links between individual differences in social dominance motivation, neural processing, and behavioral performance.*

***Page 31-32, 3.3. Correlational analyses*: *Spearman’s rho correlation and simple linear regression analyses were conducted to examine the predictive relationship between PRF-d scores and RT, FRN, P3 amplitudes, and their corresponding RPE signals. Results indicated no significant correlation between PRF-d scores and RT across any condition (all R² < 0.02, p > 0.32) (Fig. 8a).***

***However, a significant relationship emerged between PRF-d scores and the mean amplitude of FRN in several experimental conditions. Specifically, PRF-d scores significantly predicted FRN amplitude in the EXP-certain positive (R² = 0.11, F(1, 49) = 6.33, p = 0.015), EXP-certain negative (R² = 0.13, F(1, 49) = 7.51, p = 0.009), EXP-uncertain positive (R² = 0.14, F(1, 49) = 7.82, p = 0.007), EXP-uncertain negative (R² = 0.14, F(1, 49) = 8.22, p = 0.006), and UNEXP-uncertain negative (R² = 0.08, F(1, 49) = 4.57, p = 0.038) conditions. A similar trend was observed in the UNEXP-uncertain positive condition (R² = 0.06, F(1, 49) = 3.36, p = 0.073), although it did not reach statistical significance (Fig. 8b). These findings suggest that lower PRF-d scores are associated with more negative FRN amplitudes, reflecting heightened sensitivity to feedback.***

***A significant positive relationship was also found between PRF-d scores and P3 amplitude in the UNEXP-uncertain negative condition (R² = 0.09, F(1, 49) = 4.98, p = 0.030). No significant associations were observed between PRF-d scores and P3 amplitude in the remaining conditions (p > 0.07) (Fig. 8c).***

***Regarding the RPE signal derived from FRN, regression analyses revealed a significant relationship between PRF-d scores and RPE in the UNEXP-uncertain condition (R² = 0.09, F(1, 49) = 4.98, p = 0.030). Specifically, individuals with higher PRF-d scores (i.e., the high-dominance group) exhibited more negative RPEs in this condition. No significant associations were found between PRF-d scores and FRN-based RPEs in other conditions (p > 0.5) (Fig. 8d). Likewise, no significant correlations were observed between PRF-d scores and P3-based RPEs across any condition (p > 0.4) (Fig. 8e).***

***Fig 8. Correlation matrix (left), and Simple linear regressions (right). a) PRF-d score and RT; b) PRF-d score and FRN amplitude; c) PRF-d score and P3 amplitude; d) PRF-d score and RPEs of FRN amplitude; e) PRF-d score and RPEs of P3 amplitude.***

***Page 41-42, 4. Discussion, lines 912-923: The correlation findings reveal that individual differences in dominance, as indexed by PRF‑d scores, systematically modulate neural sensitivity to feedback under varying expectancy and uncertainty. Across both certain and uncertain contexts, lower dominance individuals were associated with more pronounced (i.e., more negative) FRN amplitudes, indicating that individuals with reduced feelings of power become especially vigilant to negative or unexpected outcomes. This effect was strongest in the unexpected–uncertain negative condition, suggesting that low‑dominance participants are particularly sensitive to unpredictable adverse feedback (46).***

***Moreover, higher dominance people were characterized by higher effect amplitudes of P3, again only in the unexpected–uncertain negative condition. Because P3 is most often related to the motivational significance of stimuli and the allocation of cognitive resources, this pattern may suggest that dominant individuals might engage in extra evaluative or regulatory processes in the face of unexpected negative feedback (13, 46, 88). The positive relationship between PRF‑d scores and FRN‑derived RPEs in the same unexpected–uncertain negative context underscores that higher‑dominance individuals experience relatively larger RPEs—reflecting a mismatch between expected and actual outcomes—when outcomes are both negative and unpredictable. Together, these results suggest a dual mechanism by which dominance amplifies early evaluative processing (FRN) and later motivational updating (P3 and RPE) in response to unanticipated negative events, highlighting the role of social power perceptions in shaping the neural dynamics of feedback processing.***

What are the different meanings of amplitude, latency and RPE of ERP components? The implication that independent variables affect these three dependent variable indicators should be explained in the discussion section.

***Answer****: Thank you for your insightful comment. In response to the reviewer’s comment, we clarified in the Discussion section that the amplitude, latency, and RPE of ERP components each reflect distinct aspects of cognitive processing influenced by our experimental manipulations. Comprehensive explanations are presented in the Introduction (****Page 4, 1. Introduction, lines 72-76, One of the electroencephalographic components, known as the feedback-related negativity (FRN), is a well-established neural marker associated with loss processing (9). This negative deflection is typically observed 200-300 milliseconds post-feedback onset in the frontal region, exhibits larger amplitudes in response to negative feedback compared to positive feedback (7, 9-16). Page 5, Lines 91-95: The P3 component plays a significant role in outcome evaluation and reward processing (25, 26). Following the detection of a mismatch between expected and actual results, the prediction model is updated to enhance its accuracy for future feedback. In response to reward and punishment stimuli, a positive deflection, known as P3, appears between 300 to 500 milliseconds post-feedback and is linked to this mechanism (27).)*** *and Methods sections (****Page 14-15, 2.3. EEG recording and signal pre-processing, lines 304-322: Grand averages of the six conditions were calculated after averaging artifact-free trials per participant and condition. Trials were grouped depending on expectation, certainty, and valence. Thus, there were six different conditions: (i) expected-certain positive feedback (Cue ‘one’; EXP-certain (POS)), (ii) expected-certain negative feedback (Cue ‘three’; EXP-certain (NEG)), (iii) unexpected-uncertain positive feedback (Cue ‘three’; UNEXP-uncertain (POS)), (iv) unexpected-uncertain negative feedback (Cue ‘one’; UNEXP-uncertain (NEG)), (v) expected-uncertain positive feedback (Cue ‘two’; EXP-uncertain (POS)), and (vi) expected-uncertain negative feedback (Cue ‘two’; EXP-uncertain (NEG)). Subsequently, the average number of positive from the average number of negative feedback trials for each participant were subtracted to measure RPE-related brain activity across the three levels of expectation that lead to EXP-certain, EXP-uncertain, and UNEXP-uncertain conditions (12, 22).***

***For the quantification of the FRN and P3 components, mean amplitude values were assessed 200-300ms after feedback onset at three electrodes (Fz, FCz, and Cz) for FRN amplitude and 300–500ms after feedback onset at Pz for P3 amplitude. The selection of these time windows and electrode locations was based on the literature revealing that the frontocentral electrodes show greater FRN amplitudes (10, 12, 14), and P3 amplitude increases from frontal to parietal region (13, 68). Further, latencies from feedback onset to the original waveforms' minimum (FRN) and maximum (P3) were also extracted. ). Additionally, based on the insightful feedback from the esteemed reviewer, further clarifications have been incorporated into the Discussion section.***

***Page 33-34, 4. Discussion, lines 718-729****:* ***The amplitude of the FRN and P300 reflects the strength of neural responses to feedback: FRN amplitude is typically more negative following unexpected or negative outcomes and is linked to performance monitoring (7, 15, 71), while P300 amplitude is more positive for salient or motivationally relevant stimuli, reflecting attentional allocation (13, 72). Latency, defined as the time from feedback onset to ERP peak, indexes the speed of cognitive evaluation, with shorter latencies indicating faster processing. The RPE, operationalized as the difference in amplitude between negative and positive feedback, reflects the neural coding of outcome discrepancies based on expectations, as conceptualized in reinforcement learning models (23). Our results suggest that the independent variables (e.g., cue type, dominance group) differentially affected these measures—modulating FRN and P300 amplitudes in line with feedback salience, influencing latency as an index of processing efficiency, and shaping RPE as a marker of prediction updating (e.g., (71, 72)).***

**Reviewer #2**

This study investigates the relationship between social dominance and different types of uncertainty, both at the behavioral and neural levels. The study is well-designed, and the analyses are well-executed. Below are my main and minor comments:

*We sincerely thank the reviewer for their time and thoughtful feedback on our manuscript. We have addressed the comments below and revised the manuscript accordingly. We believe these changes have strengthened the quality and clarity of the work.*

Main comments:

Since the authors use a learning task, I believe it would be helpful to examine the learning curves both preference and reaction times (RTs), “during” each block, and separately for each cue. Given the large number of trials per condition, it is possible that behavioral effects differ substantially between the early and late phases of each block. Furthermore, because this is a learning task, the authors are in a strong position to investigate whether individuals with different levels of social dominance learn differently. Therefore, I recommend analyzing behavioral data dynamically across time, not just as grand averages. The author can effectively quantify “learning rates” in their data, based on the different experimental conditions and participant groups.

***Answer:*** *We thank the reviewer for suggesting analyzing learning rate curves for accuracy and RTs within blocks, separately for cue and social dominance groups. We performed further trial-level analyses within each block to provide a more precise understanding of learning rates and time-dependent behavioral adaptation.*

*1. Learning across time in the training phase*

*a. Accuracy (Preference): We divided the 60 training trials into two equal blocks and examined accuracy for Cue 1 and Cue 2 in low‑ and high‑dominance groups. Both groups exhibited marked improvements: low‑dominance accuracy rose from 74.55% to 91.96% for Cue 1 and from 29.44% to 43.63% for Cue 2, while high‑dominance accuracy increased from 76.85% to 92.65% and from 25.72% to 40.19%, respectively. Linear regression confirmed significant positive slopes (≈14–17; ps < .01) across all four conditions, demonstrating robust learning and providing quantitative estimates of acquisition speed.*

*b. Reaction Time (RT): RTs likewise decreased over the training phase, indicating improved processing efficiency. In the high‑dominance group, RT declined significantly from 0.692 s to 0.579 s for Cue 1 (p = .002) and from 0.757 s to 0.627 s for Cue 2 (p = .008). Compared to the high-dominance group, the low dominance group seems to have manifested a greater reduction in arrest RTs for Cue 2 though this did not emerge as significant for the Cue 1 condition (from 0.746 s to 0.586 s, p = .008). This, therefore, supports the notion that high dominance reacts faster and in more consistent ways to outcome-based learning, particularly when positive reinforcement is involved.*

*2. Learning across time in the main Experiment*

*a. Accuracy (Preference): Regression analyses in experimental blocks 1 to 10 (50 trials per block) found no evidence for any general trend in accuracy (with slopes being roughly zero and ps > 0.1; R² approximately zero), suggesting that preferences stabilized early. . Nevertheless, repeated‑measures ANOVA detected a main effect of block (p = .004) and a cue × block interaction (p = .010), indicating subtle non‑linear fluctuations. For Cue 1 in the low‑dominance group, for example, significant pairwise differences between early and late blocks (e.g., Block 3 vs. 8, Δ = –10.64, p = .007; Block 5 vs. 8, Δ = –11.72, p = .001; Block 1 vs. 9, Δ = –13.4, p = .004) underscore a clear decline in accuracy over time. Cue 2 showed more modest shifts (e.g., Block 2 vs. 5 in high‑dominance: Δ = –11.74, p = .024; Block 1 vs. 10: Δ = –21.2, p = .014), while Cue 3 remained largely stable, with only two significant late‑phase differences (Block 2 vs. 10 and Block 5 vs. 10, both ps < .01), reflecting minimal change.*

*b. Reaction Time (RT): RT, however, showed clearer and more consistent improvements. Regression analyses revealed significant negative slopes in 4 out of 6 cue × group combinations, indicating progressively decreasing response time. The cue × block interaction was also significant (p =.008), further supporting cue-specific learning patterns. Post-hoc analysis (e.g., Cue 1 Block 1 vs Block 9, p = .004) demonstrated strong gains that establish RT as being more sensitive than accuracy to ongoing learning.*

*Detailed group-level RT trends support these findings:*

*Low-Dominance Group:*

*Cue 1: Started at 0.559s, fluctuated slightly, and declined to 0.450s by Block 9.*

*Cue 2: Fluctuated without a clear trend but showed high variability.*

*Cue 3: Displayed substantial fluctuations, peaking at Block 4 (0.714) and stabilizing thereafter.*

*High-Dominance Group:*

*Cue 1: Declined steadily from 0.580s to 0.420s by Block 9, with minor fluctuations.*

*Cue 2: Gradual decrease from 0.698s to 0.471s by Block 8, with a slight rebound.*

*Cue 3: Varied between 0.548s and 0.732s, showing no clear directional trend.*

*3. Group Differences in Learning Rates*

*Throughout both phases, high-dominance participants also achieved these reaction-time improvements faster and more stably, especially for Cue 1; their decreases were more uniform and less variable vis-à-vis those of the low-dominance group. Whereas, both groups had experienced similar accuracy gains on training, only the high-dominance group kept improving timewise throughout the experimental session.*

*We have integrated these additional analyses and updated figures into the revised manuscript.*

***Page 20-24, 3.1.3. Learning Rate: One behavioral consequence that can be interpreted through statistical analysis is the learning effect, particularly as reflected in changes in accuracy and RT during the training and Experiment phases across blocks. Fig 5 illustrates this trend by showing the average percentage of positive feedback, and RT shifts for each cue, separately for the low- and high-dominance groups.***

***The 60 training trials were divided into two blocks. In the low-dominance group, the mean percentage of positive feedback for Cue 1 increased from 74.55% in Block 1 to 91.96% in Block 2; for Cue 2, it rose from 29.44% to 43.62%. A similar trend was observed in the high-dominance group: Cue 1 increased from 76.85% to 92.65%, and Cue 2 from 25.72% to 40.19% (see Fig. 5a).***

***Simple linear regression analyses confirmed statistically significant learning effects in all cues in both groups, with positive slopes indicating systematic improvements (high-dominance_cue1: 15.80 (p = 0.006), cue2: 14.47 (p < 0.001); low-dominance_cue1: 17.41 (p = 0.007), and cue2: 14.19 (p < 0.001)), each with modest but meaningful R² values (ranging from 0.13 to 0.29). Therefore, accuracy improved reliably with practice. Mixed repeated-measures ANOVA further supported these findings, revealing significant main effects for cue [F_(1, 49)_ = 252.39, p < 0.001,*** $\boldsymbol{\eta}_{\boldsymbol{p}}^{\boldsymbol{2}}$ ***= 0.837] and block [F_(1, 49)_ = 97.82, p < 0.001,*** $\boldsymbol{\eta}_{\boldsymbol{p}}^{\boldsymbol{2}}$***= 0.666], but not for group [F_(1, 49)_ = 0.14, p = 0.707,*** $\boldsymbol{\eta}_{\boldsymbol{p}}^{\boldsymbol{2}}$ ***= 0.003].***

***Paired samples t-tests revealed significant improvements across blocks for both groups. In the low-dominance group, Cue 1 rose from 74.55% to 91.96% and Cue 2 from 29.44% to 43.63% (both p < 0.001). Similarly, in the high-dominance group, Cue 1 improved from 76.85% to 92.65%, and Cue 2 from 25.72% to 40.19% (both p < 0.001). These findings suggest that both groups demonstrated comparable learning trajectories. Notably, the low-dominance group showed a slightly larger improvement for Cue 1 (17.41 vs. 15.80), although they started from a marginally lower baseline. For Cue 2, both groups improved by nearly the same magnitude, though the low-dominance group began with a higher initial accuracy. Moreover, the high-dominance group displayed less variability in Block 2, indicating more consistent performance.***

***In contrast, during the experiment session, no such learning trend emerged. For the low-dominance group, mean accuracy for Cue 1 increased slightly from 68.66% to 73.72%, while Cue 2 decreased marginally from 29.66% to 29.02%, and Cue 3 remained virtually unchanged (15.45% to 15.66%). The high-dominance group showed similar trends: Cue 1 increased from 70.07% to 74.68%, while Cue 2 and Cue 3 declined slightly (31.76% to 30.33% and 15.38% to 13.75%, respectively) (Fig. 5b). Linear regression analyses yielded non-significant slopes for all six conditions, indicating no meaningful learning effect across the blocks. Slopes ranged from –0.0884 to 0.3355, with all p > 0.1, and R² values near zero, suggesting stable performance without systematic improvement.***

***A mixed repeated-measures ANOVA during the experimental session confirmed a significant main effect for cue [F_(1.24, 60.86)_ = 1492.13, p < 0.001,*** $\boldsymbol{\eta}_{\boldsymbol{p}}^{\boldsymbol{2}}$***= 0.968], with higher accuracy for Cue 1 than Cues 2 and 3, and a significant main effect for block [F_(5.71, 279.86)_ = 3.3, p = 0.004,*** $\boldsymbol{\eta}_{\boldsymbol{p}}^{\boldsymbol{2}}$***= 0.063]. However, no significant main effect for group was found [F_(1, 49)_ = 1.20, p = 0.279,*** $\boldsymbol{\eta}_{\boldsymbol{p}}^{\boldsymbol{2}}$***= 0.024]. A significant interaction emerged between cue × block [F_(6.48, 317.59)_ = 2.77, p = 0.010,*** $\boldsymbol{\eta}_{\boldsymbol{p}}^{\boldsymbol{2}}$***= 0.054], while no other interactions reached significance.***

***Post-hoc analyses of the cue × block interaction revealed selective effects. For Cue 1, several blocks showed significant differences, suggesting minor fluctuations in performance. For example, Block 1 differed significantly from Block 5 (p = 0.049), and Block 3 from Block 8 (p = 0.002). Cue 2 exhibited only one significant difference (Block 2 vs. Block 5, p = 0.040), while Cue 3 showed two (Block 2 vs. Block 10, and Block 5 vs. Block 10, p < 0.001 and p = 0.006, respectively), indicating minimal variation over time.***

***In terms of RTs during the training session, both dominance groups showed general improvements. In the low-dominance group, RTs for Cue 1 declined from 0.73 to 0.65 seconds and for Cue 2 from 0.75 to 0.59. The high-dominance group displayed similar trends, with Cue 1 decreasing from 0.69 to 0.58 seconds and Cue 2 from 0.76 to 0.63 (Fig. 5c). Regression analysis revealed negative slopes for both cue 1 and cue 2 in both groups but only cue 2 in low-dominance group was statistically significant (R^2^ = 0.1403, f_(1,44)_ = 7.181, p = 0.010), suggesting that while all conditions trended downward, only one showed a robust practice-related RT improvement.***

***Mixed repeated-measures ANOVA on training RTs revealed a significant main effect of block [F_(1, 49)_ = 22.87, p < 0.001,*** $\boldsymbol{\eta}_{\boldsymbol{p}}^{\boldsymbol{2}}$***= 0.318], indicating faster responses in the second block. There were no significant main effects for cue [F _(1, 49)_ = 0.23, p = 0.630,*** $\boldsymbol{\eta}_{\boldsymbol{p}}^{\boldsymbol{2}}$ ***= 0.005]; and group [F _(1, 49)_ = 0.08, p = 0.775,*** $\boldsymbol{\eta}_{\boldsymbol{p}}^{\boldsymbol{2}}$ ***= 0.002]. Paired sample tests confirmed significant RT reductions in the high-dominance group for both cues (Cue 1: p = 0.002; Cue 2: p = 0.008). In contrast, only Cue 2 reached significance in the low-dominance group (p = 0.008), with Cue 1’s reduction falling short (p = 0.253). This suggests more consistent and generalized learning effects in the high-dominance group. RT variability was also higher in the low-dominance group, particularly for Cue 1, potentially accounting for the weaker effect.***

***During the experimental session, RTs also showed overall reductions, indicating a speed-accuracy trade-off. In the low-dominance group, Cue 1 decreased from 0.56 to 0.47 seconds and Cue 2 from 0.66 to 0.41, while Cue 3 slightly increased. In the high-dominance group, Cue 1 dropped from 0.58 to 0.46, Cue 2 from 0.70 to 0.52, and Cue 3 from 0.69 to 0.60 (Fig. 5d).***

***Simple linear regressions for RT during the experiment revealed significant negative slopes in cue 1, and cue 2 in both groups, (Slopes ranged from –0.01192 to –0.01853, all p value < 0.0025) again indicating modest but consistent learning effects. while cue 3 in low/high-dominance group remained non-significant (all P value > 0.21). Despite low R² values (low-dominance: –0.0056, high-dominance: –0.0020), these patterns confirm that repeated practice contributed to slight but reliable reductions in RT.***

***A mixed repeated-measures ANOVA of RT during the experiment confirmed a significant main effect for cue [F_(1.74, 85.63)_ = 32.24, p < 0.001,*** $\boldsymbol{\eta}_{\boldsymbol{p}}^{\boldsymbol{2}}$***= 0.397], with Cue 1 yielding the fastest responses. The main effect for block was also significant [F(6.31, 309.52) = 5.97, p < 0.001,*** $\boldsymbol{\eta}_{\boldsymbol{p}}^{\boldsymbol{2}}$***= 0.109], highlighting that responses were generally quicker by Block 10. No significant effect for group was found. Additionally, a significant interaction between cue and block emerged [F_(8.25, 404.71)_ = 2.61, p = 0.008,*** $\boldsymbol{\eta}_{\boldsymbol{p}}^{\boldsymbol{2}}$***= 0.051], with post-hoc comparisons confirming that Cue 1 showed the clearest pattern of decline over time (Block 1 vs. Block 4: p = 0.005; Block 1 vs. Block 8: p = 0.006; Block 1 vs. Block 9: p = 0.004; Block 2 vs. Block 4: p = 0.003; Block 2 vs. Block 6: p = 0.020; Block 2 vs. Block 7: p = 0.002; Block 2 vs. Block 8: p = 0.001; Block 2 vs. Block 9: p < 0.001; Block 2 vs. Block 10: p = 0.006) particularly between early and late blocks. Cue 2 displayed a similar, though less consistent (Block 1 vs. Block 7: p = 0.008; Block 1 vs. Block 8: p = 0.026; Block 1 vs. Block 9: p = 0.005; Block 1 vs. Block 10: p = 0.014), trend. Cue 3 remained largely stable across groups and blocks, with minimal changes.***

***Fig 5: The learning effect during the training and experiment phases across blocks, particularly as reflected in changes in accuracy (percentage of positive feedback) and RT; (a) left side: average of accuracy curves for cues in train session, right side: individual accuracy curves per cue and group in train session; (b) average of accuracy curves for cues in experiment session, right side: individual accuracy curves per cue and group in experiment session; (c) left side: RT in train session, right side: individual RT curves per cue and group in train session; (d) left side: RT in experiment session, right side: individual RT curves per cue and group in experiment session. Thick lines characterize the mean across task block and shaded areas indicate the standard error of the mean (sem).***

Again, the task used in this study is a reward-based learning paradigm, which is well-studied in the reinforcement learning literature. Since here there are known neural proxies for reward prediction error (e.g., FRN, P3), it would be valuable to relate the current neural findings to RL theory. Ideally, fitting a simple RL model to the behavioral data could reveal whether learning mechanisms differ by social dominance. I understand that this may be outside the scope of the current study, and I do not consider it essential. However, at a minimum, a paragraph in the discussion exploring how the findings could be interpreted through the lens of RL theory would be very helpful. especially in light of Comment 1, which may shed light on whether social dominance is related to differences in learning processes (specifically learning rate).

***Answer:*** *We thank the reviewer for this insightful suggestion about how the reinforcement learning theory might be related to our reward-based learning paradigm. We agree that the task structure--in which participants have to learn from feedback about cue-outcome associations--closely parallels core principles of RL, especially RPE-based updating (which is frequently linked to neural signals the FRN and P3 component). Although implementing a full computational RL model is beyond the scope of the current manuscript, we have added a paragraph to the Discussion section exploring how our behavioral and neural findings can be interpreted through the lens of RL theory, particularly in relation to learning rates and social dominance differences. Although, We expanded the introduction (At the comment of the editor) in the manuscript to better highlight how our work contributes to this literature (****Page 3-8, 1. Introduction****).*

***Page 42-43, 4. Discussion, lines 931-945: Finally, our observations can profitably be interpreted in line with RL theory, which postulates that behavior is shaped by PEs (i.e., the difference between expected and actual outcome) that update the value attached to choice options through a learning rate parameter (95, 96). In this framework, FRN is widely considered to indicate a signed RPE, while the P3 component is thought to reflect the salience or associability of outcomes, scaling with the absolute magnitude of the PE (7, 52). In our study, both high- and low-dominance groups exhibited comparable behavioral learning trajectories during training—accuracy improvements and decreasing RTs—suggesting similar effective learning rates. A reflection of this is the pattern of the FRN amplitude, which was modulated by valence and expectancy of the outcome but did not differ between groups, implying matched computations of RPEs across levels of social dominance. However, the high-dominance group showed larger P3 amplitudes, especially with unexpected positive feedback, suggesting that there may be increased attentional engagement or salience attribution to rewarding outcomes. This dissociation matches RL models that argue for a separable role of RPE computation and outcome integration and suggests that dominance may modulate downstream processes related to outcome evaluation, rather than the very core error-driven learning mechanism.***

***Page 44, 4.3. Limitation, line 964-967: Although we have not actually fitted RL models here, the behavioral and ERP results could be used to support or refute hypotheses in future modeling studies. For instance, extending this interpretation further, one might fit a Q-learning or RL-DDM model (reinforcement learning drift diffusion model) to choice and ERP data to estimate separate learning rates for gains and losses.***

This study reports a neural effect of group (dominance), but no main behavioral effect; only a group × condition interaction. I think it is important to discuss this discrepancy more directly. What might contribute to the presence of group differences at the neural level, but not behaviorally? One possibility is related to Comment 1: with a large number of trials per condition, behavioral responses may converge over time, masking early-stage group differences. Examining behavior during early phases of the task may help clarify this. I don’t view this discrepancy as a major issue, but I believe the paper would benefit from a thoughtful discussion of it.

***Answer:*** *We appreciate the reviewer’s insightful observation and thoughtful suggestion. We agree that the mismatch between the neural group differences and the absence of a main behavioral effect is worth further contemplation. We’ve added this interpretation to the discussion section to better clarify potential mechanisms underlying the observed dissociation between neural and behavioral findings.*

*One possible explanation is that this dissociation reflects the inherently complex, multi-stage nature of cognitive processing. Neural signals are often sensitive to subtle shifts in attention, evaluation, or learning that may not be immediately apparent in overt behavior. In other words, just because the brain is reacting differently doesn’t always mean we’ll see a clear change in how someone responds—at least not right away. This gap between neural and behavioral measures may stem from the different temporal and mechanistic properties of the brain versus behavior. Neural activity often captures earlier stages of processing—like attention allocation or stimulus evaluation—long before a button is pressed or a response is made. It’s entirely possible for two groups to show distinct neural patterns even if their outward behavior (e.g., RTs or accuracy) ends up looking similar. For example, early neural markers often reflect the very first stages of perception and evaluation—when you’re just getting a sense of what’s happening—long before you press a button. Those initial processes could vary significantly between high- and low-dominance individuals, even if, by the time the motor command goes out, everyone ends up behaving the same. Importantly, the motor stage introduces another layer of variability. Even after a decision is formed, it still has to be translated into an action. This step can be influenced by things like motor readiness, task strategy, or even fatigue—all of which can blur group-level effects that originated earlier in the process. As a result, differences in neural signatures of learning or evaluation (like variability or timing) may not always align neatly with average behavioral outputs (van Kempen et al, 2019; Imani et al, 2020; Shenhav et al, 2017; Klein-Flügge et al, 2013).*

*As the reviewer rightly points out, the large number of trials in our paradigm could also contribute to the pattern we observe. Practice-based explanation - elaborated upon in Comment 1- is that substantial practice across many trials allows behavior to converge over time at the expense of masked group differences that were more evident early on. Our exploratory time-course analyses support this idea: participants with high social dominance showed quicker reaction-time improvements and more stable performance early in the task, while those with lower dominance gradually caught up. This resulted in a washed-out main effect when averaging across the full task.*

*Interestingly, this kind of neural-behavioral dissociation isn’t unique to our study. However, in recent studies, for example, by Mohammadi Kia et al. and Mohammadi Pour et al. (2024), this kind of dissociation was shown in which no significant difference was found between high- and low-dominance groups behaviorally, whereas electrophysiological data showed a clear divergence. difference. Also, it mirrors patterns seen in other areas, such as research on feedback processing in substance-use populations, where neural markers like FRN/P3 amplitude or medial-frontal theta clearly distinguish groups even when their overall accuracy is the same (e.g., Ghaderi et al., 2017).*

*Taken together, these findings suggest that future research should focus more closely on early or transitional phases of the task, when group differences in neural processing are most pronounced and may be more predictive of long-term learning outcomes.*

***Page 40-41, 4. Discussion, lines 879-898: It's worthwhile to mention, the mismatch between the neural group differences and the absence of a main behavioral effect is worth deeper reflection. One possible explanation is that this dissociation reflects the inherently complex, multi-stage nature of cognitive processing. Neural signals are often sensitive to subtle shifts in attention, evaluation, or learning that may not be immediately apparent in overt behavior. In other words, just because the brain is reacting differently doesn’t always mean we’ll see a clear change in how someone responds—at least not right away. This gap between neural and behavioral measures may stem from the different temporal and mechanistic properties of the brain versus behavior. Neural activity often captures earlier stages of processing—like attention allocation or stimulus evaluation—long before a button is pressed or a response is made. It’s entirely possible for two groups to show distinct neural patterns even if their outward behavior (e.g., RTs or accuracy) ends up looking similar. For example, early neural markers often reflect the very first stages of perception and evaluation—when you’re just getting a sense of what’s happening—long before you press a button. Those initial processes could vary significantly between high- and low-dominance individuals, even if, by the time the motor command goes out, everyone ends up behaving the same. Importantly, the motor stage introduces another layer of variability. Even after a decision is formed, it still has to be translated into an action. This step can be influenced by things like motor readiness, task strategy, or even fatigue—all of which can blur group-level effects that originated earlier in the process. As a result, differences in neural signatures of learning or evaluation (like variability or timing) may not always align neatly with average behavioral outputs (90-93).***

Minor comments:

I found the description of how dominance is determined based on the PRF_d scores a bit unclear. The text mentions that extreme values (0 and 16) were extracted using data from the remaining 322 subjects. However, the authors report the mean PRF_d scores for both groups, and these values are not exactly 0 or 16. Clarification on this criterion would be helpful.
***Answer:*** *Thank you for highlighting this point. You are correct that our original wording was ambiguous. Although the PRF‑d scale ranges from 0 to 16, we did **not** recruit only those participants who scored exactly 0 or 16. Instead, we administered the PRF‑d to 322 individuals and rank‑ordered their scores. We then selected the 30 lowest scorers (low‑dominance group) and the 30 highest scorers (high‑dominance group). To reach our target sample size while maintaining clear separation between groups, we included scores within a small range around each extreme (e.g., within one standard deviation of the cutoffs). As a result, the final group means (30 high‑dominance, 30 low‑dominance) were not exactly 0 and 16. Those 60 participants were subsequently invited to the EEG experiment. We will revise the manuscript to make it clear that selection was based on extreme values, but that the reported group means reflect a small range around those extremes rather than only the theoretical minimum and maximum. We appreciate the opportunity to clarify this point.*

***Page 9, 2.1. Participants, lines 193-200: The following, the remaining 322 participants' scores were rank ordered and were divided based on their scores in the PRF-d into two groups of low and high dominance (S1 fig) (44, 63). We then selected the 30 lowest scorers (low dominance group) and the 30 highest scorers (high dominance group). Although the PRF‑d scale theoretically ranges from 0 to 16, our final groups did not include participants at those absolute extremes; to reach our target sample size while preserving clear separation, we included scores within a small range around each endpoint_ one standard deviation above or below the cutoff. These 60 participants were subsequently invited to take part in the EEG experiment.***

Do we have access to the distribution of PRF_d scores across the 322 subjects? It would be useful to see what proportion of the population was selected and based on what distribution. This would provide a clearer picture of how the authors defined dominance relative to the general population.
***Answer:*** *Thank you for this important observation. We agree that providing information on the distribution of PRF_d scores among the 322 participants enhances the clarity and interpretability of our group selection criteria.*

*First, we described the entire PRF_d distribution for all 322 participants. Then we went ahead and defined our final sample from those at the extremes: 1-7 belonged to the low-dominance group, and 11-16 to the high-dominance group. To define “low” and “high” dominance groups, we first identified the extreme quartiles of the distribution, inviting those in the lowest quartile and the highest quartile to participate. We then continued inward from each tail—selecting additional individuals with the next-lowest and next-highest scores—until we reached our target of 30 low‑dominance and 30 high‑dominance participants.*

| ***Score*** | ***Frequency (n=322)*** | ***% (n=322)*** | ***Frequency (n=51)*** | ***% (n=51)*** |
| --- | --- | --- | --- | --- |
| *1* | *5* | *1.6%* | *0* | *0.0%* |
| *2* | *7* | *2.2%* | *1* | *2.0%* |
| *3* | *9* | *2.8%* | *1* | *2.0%* |
| *4* | *14* | *4.3%* | *4* | *7.8%* |
| *5* | *20* | *6.2%* | *5* | *9.8%* |
| *6* | *29* | *9.0%* | *7* | *13.7%* |
| *7* | *31* | *9.6%* | *5* | *9.8%* |
| *8* | *33* | *10.2%* | *0* | *0.0%* |
| *9* | *0* | *0.0%* | *0* | *0.0%* |
| *10* | *27* | *8.4%* | *0* | *0.0%* |
| *11* | *31* | *9.6%* | *8* | *15.7%* |
| *12* | *32* | *9.9%* | *6* | *11.8%* |
| *13* | *26* | *8.1%* | *7* | *13.7%* |
| *14* | *20* | *6.2%* | *3* | *5.9%* |
| *15* | *19* | *5.9%* | *3* | *5.9%* |
| *16* | *8* | *2.5%* | *1* | *2.0%* |

*By juxtaposing the full 322-participant distribution against the 51-person subsample, we can see exactly what slice of the population was recruited and how “low” and “high” dominance map onto the empirical PRF-d curve. In the full cohort, scores 1–7 together account for 35.7% of participants, yet they represent 45.1% of our subsample (0% at 1, 2% at 2–3 each, then rising through 7). Conversely, scores 11–16 make up 42.2% of the full distribution but 55.0% of those selected (peaking at 15.7% for score 11 and tapering to 2% at 16). Meanwhile, the mids (8–10), which constitute 18.6% of the full sample, are entirely absent from the subsample. This enrichment of the bottom and top tails—and the complete exclusion of central scores—demonstrates that our “low” and “high” groups were explicitly defined as the extreme \~10–15% tails of the observed PRF-_d distribution, rather than arbitrary cutoff points.*

 ***S1 fig. PRF-d distribution***

Additionally, I believe it would be helpful if the authors reported the percentage of trials, ICA components, and electrodes that were removed or interpolated. Including this information in the methods section would improve transparency and reproducibility.

***Answer:*** *Thank you for this helpful suggestion. We have added the following details to the Methods to improve transparency:*

***Page 14, 2.3. EEG recording and signal pre-processing, lines 291-298: Using EEGLAB 2021.1, the raw EEG signals were first filtered with a windowed FIR sync filter to remove line noise and other artifacts by applying a high-pass filter to frequencies above 0.3Hz and a low-pass filter to frequencies below 30Hz. Next, the EEG recordings were inspected visually to detect artifacts. Epochs with artifacts were thrown out and not used for further processing. Eye blinks, muscular tension and lousy channel artifacts were identified and removed through independent component analysis (ICA) (For each participant, the percentage of epochs that were rejected is below 10%.). Finally, no channels were identified as consistently defective, and therefore, no electrodes required interpolation, and EEG signals were prepared for ERP processing.***
