# Supplementary material for: The interplay between social dominance and decision-making under expected and unexpected uncertainty: Evidence from event-related potentials
Source: PLoS One. 2025 Oct 17;20(10):e0334065. doi: 10.1371/journal.pone.0334065 (PMC12533924; doi:10.1371/journal.pone.0334065)
Supplement: Attachment — Submitted filename: Response to reviewer 8 26 2025.docx [file pone.0334065.s003.docx]

**Response to Editor and reviewer:**

We sincerely thank the editor and reviewers for the time and effort they invested in evaluating our manuscript. We believe that the revisions made in response to their feedback have substantially improved the quality of the work. In this document, the reviewers’ original comments are presented in standard font and dark blue, while our responses are provided in *black italics*. Where relevant, revisions to the manuscript are highlighted in **bold**.

**Editor**
the data repository requires attention. First, the stated link (https://osf.io/4rsmg/) is inactive. Second, the repository lacks a metadata file (e.g., readme.txt) that describes its contents. Such a file should be included for clarity and transparency. Third, the repository currently contains only summary data. If feasible, the authors are encouraged to also upload the raw data to enhance reproducibility and data integrity.

**Answer:** *Thank you for the reminder*, *we acknowledge the issues with the OSF repository:*

*The previously provided link* ***(https://osf.io/4rsmq/)****is now corrected and active.*

*A `****readme.txt`*** *file has been added to describe the contents of the repository, including the descriptions, summary data and associated materials. In addition to summary, we have uploaded the behavioral and neural datasets, enhancing reproducibility and data transparency.*

*We believe these updates fully address the editor’s concerns regarding accessibility, metadata clarity, and reproducibility.*

**Reviewer #2:**

 Thanks to the authors for providing a revised version. I believe they have done a great job in addressing the concerns. Here, I am pointing out some minor issues that I suggest should be addressed.

*Thank you very much for your positive valuation of our revised manuscript. We truly appreciate your constructive feedback and will carefully address the minor issues you have pointed out.*

(1) Take a look at these two sentences:

participants with high social dominance showed quicker RT improvements and more stable performance early in the task, while those with lower dominance gradually caught up.

In our study, both high- and low-dominance groups exhibited comparable behavioral learning trajectories during training—accuracy improvements and decreasing RTs—suggesting
similar effective learning rates.

I think this may be somewhat contradictory. Based on the reported statistics, it appears that there is no effect of group in learning, which is interesting. However, this could have been communicated more clearly.

***Answer:*** *We appreciate the reviewer’s careful reading and insightful comment. We agree that the way these two sentences were phrased could create the impression of a contradiction. Our intent was to distinguish between two different levels of analysis:*

*Exploratory time-course analysis (****early phase****): These analyses suggested that high-dominance participants appeared to show quicker RT improvements and more stable performance during the initial stages of training, whereas low-dominance participants caught up gradually. This was meant to highlight transient trends rather than group-level differences that persisted across the full task.*

*Confirmatory statistical analysis (****whole trajectory****): The mixed ANOVA and regression analyses showed* ***no significant main effect of group*** *and broadly comparable accuracy and RT improvements across dominance levels. In other words, though descriptive trends from the early part of an experiment suggested that perhaps the high-dominance group stabilized performance faster than their counterpart, differences in learning rates measured over the full course of training were not statistically distinguishable.*

*Therefore, to prevent confusion, we revised the text to make this distinction explicit.*

***Page 41:*** *Moreover, the large number of trials in our task could also contribute to this pattern. Practice-based explanation is that extensive practice across many trials allows behavior to converge over time, potentially masking group differences that were more apparent early on. Our exploratory time-course analyses support this idea:* ***Specifically, descriptive trends suggested that participants with high social dominance appeared to show quicker RT improvements and more stable performance early in the task, while those with lower dominance gradually caught up. However, by the end of training, these transient differences had diminished, resulting in no reliable group effect when performance was averaged across the full task.***

***Page 42****: Finally, our observations can profitably be interpreted in line with RL theory, which postulates that behavior is shaped by PEs (i.e., the difference between expected and actual outcome) that update the value attached to choice options through a learning rate parameter (95, 96). In this framework, FRN is widely considered to indicate a signed RPE, while the P3 component is thought to reflect the salience or associability of outcomes, scaling with the absolute magnitude of the PE (7, 52).* ***In our study, both high- and low-dominance groups ultimately exhibited comparable behavioral learning trajectories during training—accuracy improvements and decreasing RTs—indicating similar effective learning rates across groups.***

(2) I think the authors argue in several places in the manuscript that the discrepancy between neural and behavioral data could be due to variables that are more easily detectable in neural data but less so in behavioral data. I agree with this interpretation. One proposed candidate is attention. The neural data equivalent of RL models would also point to a similar candidate. In many perceptual decision-making tasks, however, attention is both behaviorally and computationally measurable, for example through differences in reaction time and/or accuracy, and through drift rate in the drift diffusion model (DDM). Overall, I think this study suggests that attention differs between dominance groups. This is both experimentally and computationally testable via tasks that are more sensitive to attention change than the current task. Therefore, I suggest that the authors make this point critically clear and encourage readers to test the findings of the current work in future studies.

***Answer:*** *We thank the reviewer for this thoughtful comment and fully agree that attention is a strong candidate mechanism underlying the neural–behavioral dissociation we observed. As noted, while our task allowed us to identify group differences in FRN and P3 amplitudes, the behavioral measures in this paradigm (accuracy, RT) may not have been sensitive enough to capture delicate attentional effects. We appreciate the reviewer’s suggestion to highlight that attention can be experimentally and computationally measured in other paradigms (e.g., drift diffusion modeling of drift rate) and to encourage future work to test our findings using tasks optimized for attention. We have now clarified this in the limitations section, emphasizing attention as a promising avenue for further empirical and modeling-based investigation.*

***Page 44, limitation:*** ***Another*** ***limitation of the present study is that the task was not optimized to directly assess attentional mechanisms. Although the neural findings suggest group differences in attentional engagement, our behavioral indices (accuracy, RT) may not have sensitivity comparable to neural measures and thus failed to reflect such effects. Future work could employ paradigms specifically designed to probe attention, such as Posner cueing or sustained attention tasks or tasks that are more sensitive to attention, in combination with computational modeling approaches like the DDM, where drift rate provides a quantitative marker of attentional allocation and evidence accumulation (8, 98-102). Such approaches would allow a more precise test of whether social dominance modulates reinforcement learning via attentional mechanisms, thereby extending the neural–behavioral dissociation reported here.***

(3) This sentence has been appended to the end of a paragraph related to learning, whereas I think it should have been appended to the one discussing the discrepancy between behavioral and neural data.

Interestingly, this kind of neural-behavioral dissociation isn’t unique to our study. In previous
works, such as those by (94), this pattern was also observed, where no significant difference was found in behavioral observations between the high and low dominance groups, whereas
electrophysiological data showed a clear difference. Also, it mirrors patterns seen in other areas,
such as research on feedback processing in substance-use populations, where neural markers like FRN/P3 amplitude or medial-frontal theta clearly distinguish groups even when their overall
accuracy is the same (e.g., (17)).

***Answer:*** *We appreciate the comment and agree that the sentence fits better with the discussion of the discrepancy between behavioral and neural data, and we've moved it accordingly.*

***Page 40:*** *It's worthwhile to mention, the mismatch between the neural group differences and the absence of a main behavioral effect is worth deeper reflection.* ***Interestingly, this kind of neural-behavioral dissociation isn’t unique to our study. In previous works, such as those by (93), this pattern was also observed, where no significant difference was found in behavioral observations between the high and low dominance groups, whereas electrophysiological data showed a clear difference. Also, it mirrors patterns seen in other areas, such as research on feedback processing in substance-use populations, where neural markers like FRN/P3 amplitude or medial-frontal theta clearly distinguish groups even when their overall accuracy is the same (e.g., (17)).*** *One possible explanation is that this dissociation reflects the inherently complex, multi-stage nature of cognitive processing. Neural signals are often sensitive to subtle shifts in attention, evaluation, or learning that may not be immediately apparent in overt behavior.*
